# Mapping the expression of transient receptor potential channels across murine placental development

**DOI:** 10.1007/s00018-021-03837-3

**Published:** 2021-04-21

**Authors:** Katrien De Clercq, Vicente Pérez-García, Rieta Van Bree, Federica Pollastro, Karen Peeraer, Thomas Voets, Joris Vriens

**Affiliations:** 1 Laboratory of Endometrium, Endometriosis and Reproductive Medicine, Department of Development and Regeneration, KU Leuven, Herestraat 49, Box 611, 3000 Leuven, Belgium; 2 Laboratory of Ion Channel Research, Department of Cellular and Molecular Medicine, VIB Center for Brain and Disease Research, KU Leuven, Herestraat 49, Box 802, 3000 Leuven, Belgium; 3 Department of Physiology, Development and Neuroscience, Centre for Trophoblast Research, University of Cambridge, Downing Street, Cambridge, CB2 3EG UK; 4 Epigenetics Programme, The Babraham Institute, Babraham Research Campus, Cambridge, CB22 3AT UK; 5 Centro de Investigación Principe Felipe, Molecular Basis of Human Diseases, Eduardo Primo Yúfera 3, 46012 Valencia, Spain; 6 Dipartimento di Scienze del Farmaco, Universita degli Studi del Piemonte Orientale Amedeo, Vercelli, 13100 Italy

**Keywords:** TRP channels, Placental development, Primary trophoblast cells, Trophoblast stem cells

## Abstract

Transient receptor potential (TRP) channels play prominent roles in ion homeostasis by their ability to control cation influx. Mouse placentation is governed by the processes of trophoblast proliferation, invasion, differentiation, and fusion, all of which require calcium signaling. Although certain TRP channels have been shown to contribute to maternal–fetal transport of magnesium and calcium, a role for TRP channels in specific trophoblast functions has been disregarded. Using qRT-PCR and in situ hybridisation, the spatio-temporal expression pattern of TRP channels in the mouse placenta across gestation (E10.5–E18.5) was assessed. Prominent expression was observed for *Trpv2*, *Trpm6*, and *Trpm7*. Calcium microfluorimetry in primary trophoblast cells isolated at E14.5 of gestation further revealed the functional activity of TRPV2 and TRPM7. Finally, comparing TRP channels expression in mouse trophoblast stem cells (mTSCs) and mouse embryonic stem cells (mESC) confirmed the specific expression of TRPV2 during placental development. Moreover, TRP channel expression was similar in mTSCs compared to primary trophoblasts and validate mTSC as a model to study TRP channels in placental development. Collectivity, our results identify a specific spatio-temporal TRP channel expression pattern in trophoblasts, suggesting a possible involvement in regulating the process of placentation.

**Supplementary Information:**

The online version contains supplementary material available at 10.1007/s00018-021-03837-3.

## Introduction

Despite its transient existence, the placenta is imperative for fetal survival and lifelong health [1, 2]. It accommodates cells of allogeneic origin and combines multifaceted functions that are separated in the adult [3, 4]. In mice, placental development starts with the invasion of the embryo in the maternal decidua, orchestrated by cells of the trophectoderm, a layer of epithelial-like cells surrounding the blastocyst. Cells from the polar trophectoderm will continue to proliferate and eventually form the extraembryonic ectoderm and the ectoplacental cone. Ultimately, terminally differentiated cells from the extraembryonic ectoderm will constitute the trophoblasts of the labyrinth, separating maternal blood spaces from fetal capillaries, i.e. syncytiotrophoblasts layer-I, -II (SynT-I and SynT-II), and sinusoidal trophoblast giant cells (TGC). The ectoplacental cone composes cells that will develop into the junctional zone, namely spongiotrophoblast, glycogen cells, and various types of TGCs. Thus, placental development is a strictly coordinated process of continuous proliferation and differentiation culminating during a critical period. The mature murine placenta consists of three clearly distinguishable layers: the maternal decidua, the junctional zone that fulfils an endocrine role, and the labyrinth where nutrient exchange takes place [5].

Calcium ions affect nearly all aspects of cellular homeostasis, as one of the most important secondary messengers. Underlying its effectiveness is the vast concentration gradient between intra- (~ 100 nM) and extracellular (~ 1.5 mM) concentrations. Maintaining this steep gradient is, therefore, paramount for cells to react to transient rises in intracellular calcium. The intracellular calcium levels are controlled by a tight regulation of calcium entry, extrusion, compartmentalization, and chelation. Given that trophoblast cells are non-excitable cells, calcium entry will mainly be governed by calcium permeable channels that are activated by other mechanisms than voltage. Members of the transient receptor potential (TRP) superfamily are excellent candidates to regulate calcium influx [6]. They represent a group of 28 genes that encode for non-selective transmembrane cation channels, divided into six subfamilies in mammals according to sequence homology: TRPA, TRPV, TRPM, TRPC, TRPP, and TRPML [7]. Over the last decade, TRP channel function has emerged in a variety of processes, emphasizing their importance in normal physiology. However, their role in placental development remains largely unexplored.

Trophoblast cells are subjected to continuous and large amounts of various hormones and growth factors, be it by bathing in maternal blood, or by paracrine signaling from neighbouring cells [8, 9]. Activation or modulation by hormones and growth factors has been described for several TRP channels [10, 11]. In addition, diverse subtypes of specialized trophoblast cells emerge during development and differentiate to acquire specific functions such as invasion of the decidualized endometrium and maternal vascular remodelling, cell fusion, and hormone synthesis and secretion, all of which are instrumental for proper placental functioning. Importantly, calcium signaling is a key determinant in all these processes [12–16]. Moreover, calcium ions are essential during development not only for maintaining normal cell physiology, but also for fetal bone formation and the development of excitatory cells of the nervous system and heart [17].

In pursuit of identifying members of the TRP superfamily involved in placental physiology, the spatio-temporal expression pattern of TRP channels was defined in the mouse placenta. Temporal RNA expression was assessed on whole placental tissues from embryonic day (E) 10.5–E18.5. Given that differences in phenotype penetrance have been observed in the generation of knockout mice [18], the expression pattern was compared between the most commonly used mice strains, e.g. C57BL6/J and mixed B6/129S. Spatial expression was assessed using fluorescent in situ hybridisation (FISH) at E10.5, E14.5, and E18.5. Functional expression of TRP channels was determined in primary trophoblast cells isolated at E14.5 via calcium microfluorimetry. Finally, the molecular and functional expression was evaluated in mouse trophoblast stem cells (mTSC), as model to study TRP channels during early gestation.

## Materials and methods

### Animals

8 to 12 weeks old mice (C57BL/6J—Janvier, France, and B6/129S—internal breeding) were housed in filter-top cages under conventional conditions (23 ± 1.5 °C, relative humidity 40–60%, 12:12 light/dark cycle). Animals were mated and the detection of a copulation plug was dated as E0.5 of gestation. Placentas were collected at different days during gestation. Placentas and/or mechanically separated placental layers were preserved in RNALater buffer (Qiagen) or fixed in 4% PFA. The ethical review committee for animal experiments at the KU Leuven (Belgium) approved the use of mice for this study.

### Cell culture

#### Murine trophoblast isolation and culture

Primary trophoblast cells were isolated as described previously [19]. Briefly, placentas were collected at E14.5 or E18.5 and were dissected into small pieces before incubation in 1X Medium-199 (Sigma) containing 1 mg/mL collagenase (Sigma), 2% Penstrep, and 20 µg/mL Dnase I (Sigma). Incubation was performed for 30 min at 37 °C, wherein the tissue was pipetted every 10 min to ensure maximal enzymatic and mechanical degradation. The suspension was filtered through a 100 μM strainer (BD Falcon, Fisher Scientific) and centrifuged for 5 min (500*g*, 4 °C). The pellet was resuspended in trophoblast medium containing of NCTC-135 medium (Sigma) supplemented with 20 mM HEPES, 25 mM NaHCO_3_, 1.65 mM cysteine, 50 U/mL penicillin, 50 μg/mL streptomycin, and 10% fetal bovine serum (FBS, Gibco). The cells were layered on top of a Percoll (Sigma) gradient (60%, 40%, and 20%) and centrifuged for 20 min (2000*g*, at 4 °C). The 40% Percoll layer containing trophoblast cells (density 1.052 g/mL) was collected, suspended in trophoblast medium, and centrifuged for 5 min (500*g*, at 4 °C). The resulting cell pellet was resuspended in trophoblast medium and seeded in either plastic multiwells or collagen-coated glass coverslips (collagen I from rat tail, 50 μg/mL in 0.02 M acetic acid, BD biosciences). The cells were incubated at 37 °C, 5% CO2.

#### Mouse trophoblast stem cells (mTSCs)

Mouse trophoblast stem cells (TS-Rs26 [20], kind gift of the Rossant lab, Toronto, Canada), were cultured in 30% RPMI 1640 with l-glutamine (ThermoFisher Scientific) supplemented with 20% FBS, 1 mM sodium pyruvate (Thermofisher Scientific), 1X anti-mycotic Antibiotic (Thermofisher Scientific), 50 μM 2-mercaptoethanol (Gibco), 25 ng/mL basal Fibroblast growth factor (bFGF, Sigma) and 1 μg/mL Heparin; and 70% of conditioned medium (CM) from Mouse Embryonic Fibroblasts. Differentiation was induced by omitting bFGF, Heparin, and CM from the media.

#### qRT-PCR experiments

Quantitative RT-PCR (qRT-PCR) experiments were performed on RNA isolated from whole uterine tissues, primary trophoblast cells or mTSC cultures. RNA quantity was checked with the Thermo Scientific NanoDrop 1000 Spectrophotometer; the RNA quality of tissues was determined with the Experion RNA Analysis kit (Bio-rad) and only samples with an RNA quality indicator (RQI) > 7 were included for cDNA synthesis.

Placental tissues: The tissue, kept in RNAlater, was homogenized by use of a power homogenizer (Polytron). Total RNA was extracted with TriPure Isolation Reagent (Roche) and subsequently used for cDNA synthesis using the High-Capacity cDNA Reverse Transcription Kit (Life Technologie). Housekeeping genes = *Actb* and *Gapdh*.

Cell cultures: The RNeasy Mini Kit (Qiagen) was used according to manufacturer’s guidelines. cDNA was synthesized with Ready-To-Go You-Prime First-Strand Beads (GE Healthcare Life Sciences). Housekeeping genes = *Actb* and *Sdha* (mTSC) or *Actb* and *Gapdh* (primary trophoblasts).

Triplicate cDNA samples (2.5 × diluted) from each independent preparation were used in the StepOne PCR system (Applied Biosystems, Life Technology) using specific TaqMan gene expression assays for all TRP channel [21]. Relative expression was shown as 2^−ΔCt^ (mean ± SEM) in which ΔCt = Ct_TRP channel_ − Ct_geometric mean of endogenous controls_. Categorization of TRP channels expression as high, moderate or low was done by normalizing to TRPM7. Note that the average expression of TRPM7 of all gestational days was used as normalization value. Fold change was shown as 2^−ΔΔCt^ and was normalized to either TRPM7 expression (ΔΔCt = ΔCt_TRP channel_ − ΔCt_T mean of gestational TRPM7_) or normalized to E10.5 of gestation (ΔΔCt = ΔCt_TRP channel_ − ΔCt_TRP channel at E10.5_).

#### RNA-seq analysis

mESC and mTSC RNA-seq data were retrieved from GSE62149 [22] and PRJNA298763 [23], respectively. Data were quantified using the RNA-seq quantitation pipeline in SeqMonk (http://www.bioinformatics.babraham.ac.uk), and normalized according to total read count (reads per kilobase of transcript per million mapped reads, RPKM). Heat maps were generated using SeqMonk.

#### Fluorescence in situ hybridisation

Fluorescence in situ hybridisation (FISH) was performed following the manufacturer’s instructions of the RNAscope Multiplex Fluorescent v2 kit (ACDbio), allowing for detection of single mRNA molecules [24]. Briefly, sections (4 μM) were deparaffinized, treated with hydrogen peroxide, target retrieval agent, and protease plus. Sections were incubated for 2 h at 40 °C with specific RNAscope probes against murine TRPV2, TRPV4, TRPV6, and TRPM4, Cdh1 (E-Cadherin) Tpbpa, Prl2c2 (Proliferin), Prl3d1 (PL-1), and Prl3b1 (PL-2). Signal was amplified and detected by sequential treatment of HRP, fluorophore and HRP blocker. Ultimately, DAPI was used to stain the nuclei and slides were mounted with ProLong Gold Antifade Mountant, Images were taken using the Nikon Fluorescence microscope (CIE i) taking care to use the same exposure and gain setting for each type of staining. Staining was performed on 2–5 placentas from two independent litters.

#### Immunocytochemistry

Primary trophoblasts were fixed with 4% formaldehyde for 10 min, permeabilized with 0.2% Triton X-100 for 10 min, and blocked with 5% goat serum for 2 h. Primary antibodies were incubated overnight at 4 °C in 0.5% goat serum: monoclonal rabbit anti-human vimentin (1:500, Cell Signalling Tech, D21H3) and monoclonal mouse anti-human pan-cytokeratin (1:1000, Sigma). The secondary antibodies (1:1000 in 0.5% goat serum, AlexaFluor488-conjugated anti-mouse IgG, and AlexaFluor594-conjugated anti-rabbit IgG) were applied for 1 h at room temperature. Triple washing with PBS was performed between each step. Finally, the coverslips were mounted in medium containing DAPI (Vectashield, Vector Laboratories). Omission of the primary antibodies served as a negative control.

### Functional measurements

#### TRP pharmacology

Functional TRPV2 activity was assessed by the application of 50 µm Δ9-tetrahydrocannabinol [25] (THC, kindly provided by G. Appendino and F. Pollastro) and responses were challenged with 2 µm of the nonspecific TRPV inhibitor ruthenium red [26, 27] (RR, Sigma). The functionality of TRPV4 was evaluated by stimulation with 10 or 20 nM GSK016790A [28] (GSK, Sigma). TRPC1/4 activity was assessed by the application of 250 nM (−)-Englerin A [29] (EA, Phytolabs). TRPM7 was evaluated using 200 µm mibefradil [30] (Mib, Sigma).

#### Calcium microfluorimetry

The measurement of intracellular Ca^2+^ was performed as previously described [21]. Briefly, cells were incubated with 2 µm Fura-2 acetoxymethyl ester for at least 30 min at 37 °C. Fluorescent signals were evoked during alternating illumination at 340 and 380 nM on a Nikon Eclipse Ti fluorescence microscope. Absolute calcium concentrations were calculated from the ratio of the fluorescence signals at both wavelengths (F340/F380) after correction for the background fluorescence signals. The standard solution contained (in mM) 150 NaCl, 2 CaCl_2_, 1 MgCl_2_, 10 d-glucose, and 10 HEPES (pH 7.4 with NaOH). The solution for experiments without extracellular calcium was (in mM): 150 NaCl, 1.5 MgCl_2_, 10 d-glucose, 10 HEPES, and 5 EGTA (pH 7.4 with HCl). Ionomycin (2 µm, Sigma) was applied at the end of every experiment as a positive control.

Cells were considered responders if the calcium influx during agonist application exceeded 50 nM and when the highest value of the derivative of the calcium trace during the application of an activator exceeded at least three times the standard deviation of the derivative during basal conditions.

#### Data analysis

Data display and statistical analysis was performed using Graphpad Prism 8.4.3 (Graphpad software incorporated). Normality was tested using the D’Agostino–Pearson omnibus test. Results were considered to be statistically significant when *p* < 0.05. Statistical tests are given in figure legends.

## Results

### Temporal expression pattern of TRP channels during gestation

**Fig. 1:**
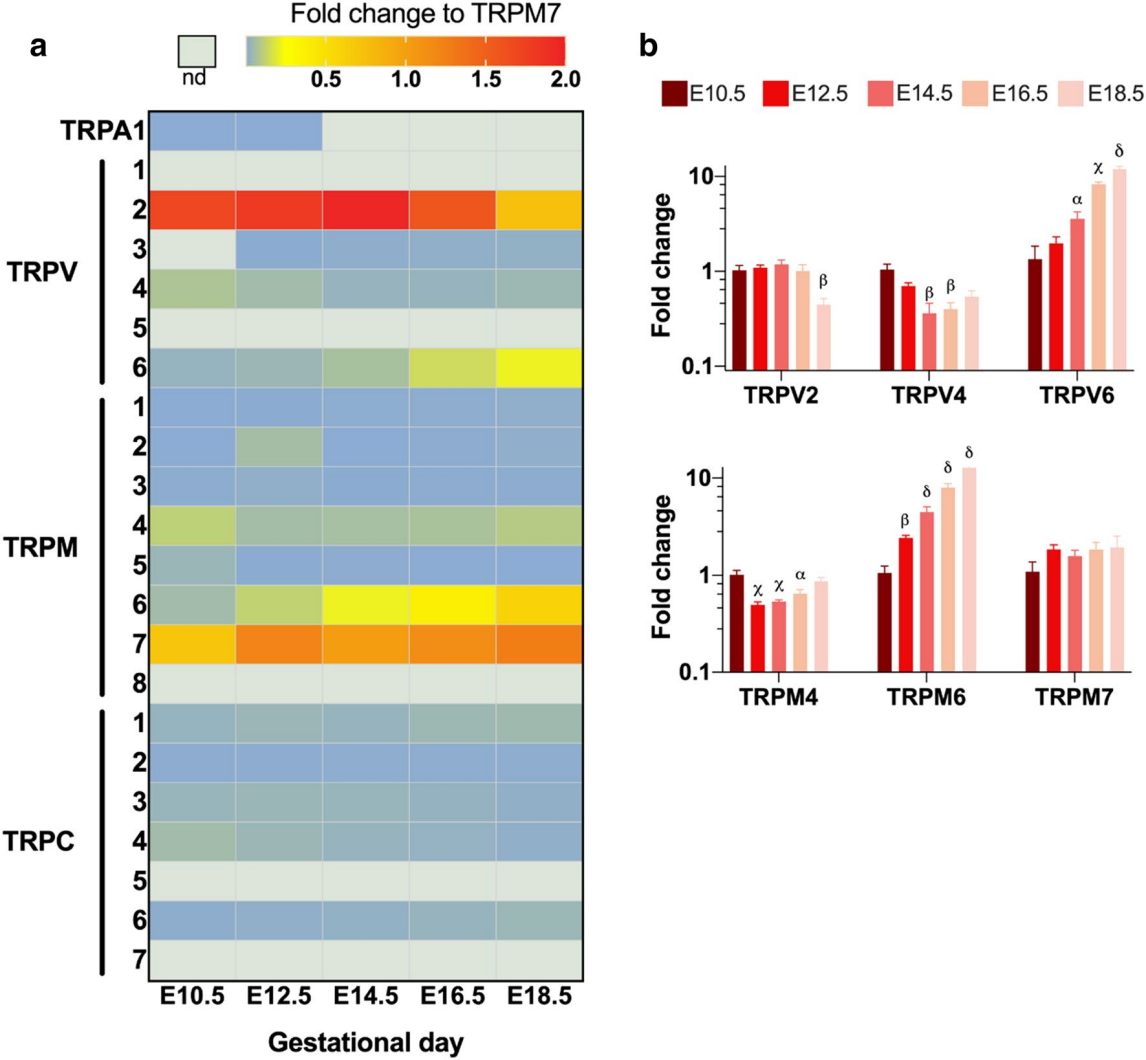
Quantitative RT-PCR showing TRP channel expression in placentas from C57BL/6 mice through gestation. **a** Heat map of mRNA levels of TRP channels relatively quantified to the geometric mean of housekeeping genes *Actb* and *Gapdh* and then normalized to the average *Trpm7* expression of all gestational days. *nd* non detectable. **b** Normalized fold change of expressed TRP channel compared to expression at E10.5, shown as mean ± SEM. Significant differences in mRNA expression were assessed with one-ANOVA followed by Dunnett’s multiple comparison test or Kruskal–Wallis test followed by Dunn’s multiple comparisons test compared to E10.5, using DeltaCT values. *α*: *p* < 0.05, *β*: *p* < 0.01, *γ*: *p* < 0.001, *δ*: *p* < 0.0001. *E* embryonic day. *n* = 4 placentas from three different litters

Using qRT-PCR analysis, the expression profile of members of the TRPA, TRPV, TRPM, and TRPC subfamilies was determined in intact placental tissues from C57BL/6 mice, harvested at different time points of gestation (Fig. 1a). TRP channel expression was categorized relative to the average *Trpm7* expression. TRPM7 is considered a housekeeping TRP channel as it is abundantly expressed and functional in nearly all cell types [31], and was found to be unchanged throughout gestation. *Trpv2* showed the highest expression (> *Trpm7* expression), compared to the moderately expressed *Trpv4*, *Trpv6*, *Trpm4*, and *Trpm6* (5–100% of *Trpm7* expression). In contrast, *Trpc1*, *Trpc3*, *Trpc4*, and *Trpc6* expression was low (1–5% of *Trpm7* expression), while the mRNA levels of *Trpa1*, *Trpv1*, *Trpv5*, *Trpm1*, *Trpm2*, *Trpm3*, *Trpm5*, *Trpm8*, *Trpc2*, *Trpc5*, and *Trpc7* were very low or below the detection level (< 1% of TRPM7 expression). TRP channels with high/moderate expression were considered for further analysis (Table 1; Fig. 1b). *Trpv6* and *Trpm6* were significantly upregulated, with more than tenfold increase towards term, whereas *Trpv2* and *Trpv4* expression decreased significantly compared to E10.5 (Fig. 1b).

**Table 1:**
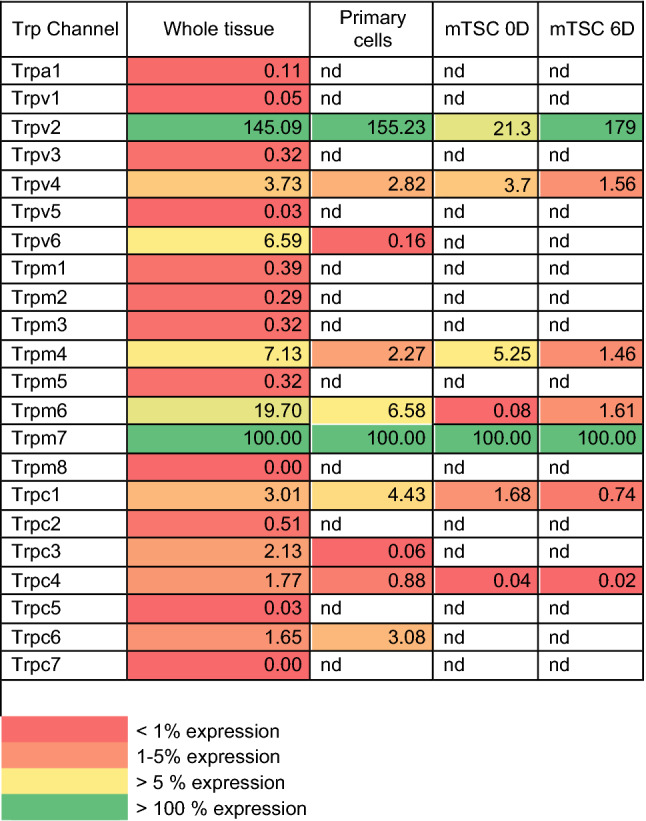
Expression levels of TRP channels in placenta shown as percentage compared to Trpm7

Placental morphology, gene expression, and phenotypes were shown to be strain-specific [32–34]. Therefore, the mRNA expression of TRP channels was compared to placentas obtained from mice with a mixed B6/129S background. A similar overall temporal expression pattern was observed for *Trpv2* and *Trpv4* showing significant decreases towards term, and for *Trpv6* and *Trpm6*, which were significantly increased in time (Supplementary Fig. 1). To further determine strain-dependent differences, the expression of each gene was compared between strains for all gestational days. Overall higher *Trpm4* expression (+19 ± 6%) and lower *Trpm6* expression levels (− 15 ± 12%) were observed in C57BL6 placentas, independent of gestational day. Further post-hoc analysis revealed significantly lower expression levels of *Trpm6* in C57BL6 placentas at E18.5 compared to B6/129S (− 33%). Taken together, these results provide a temporal overview of TRP channels in the placenta (Supplementary Fig. 2a).

### Spatiotemporal expression pattern of TRP channels in the placenta

The murine placenta exists of three distinguishable layers, e.g. the maternal decidua, the junctional zone, and the labyrinth. FISH was used to gain insight into the specific spatial expression pattern of TRP channels that were highly to moderately detected in whole placenta (Fig. 1). Note that *Trpm6*, expressed in the labyrinth [35, 36], and *Trpm7*, ubiquitously expressed in almost all placental cell types [37], were not evaluated as these results were already shown in earlier reports. Thus, for channels TRPV2, TRPV4, TRPV6, and TRPM4, the spatial expression was assessed in the premature placenta (E10.5), the mature placenta (E14.5) and term placenta (E18.5). The results obtained at E14.5 were validated by qRT-PCR on mechanically separated placental layers (Supplementary Fig. 2b, c).

### High TRPV2 expression in the labyrinth during development

**Fig. 2:**
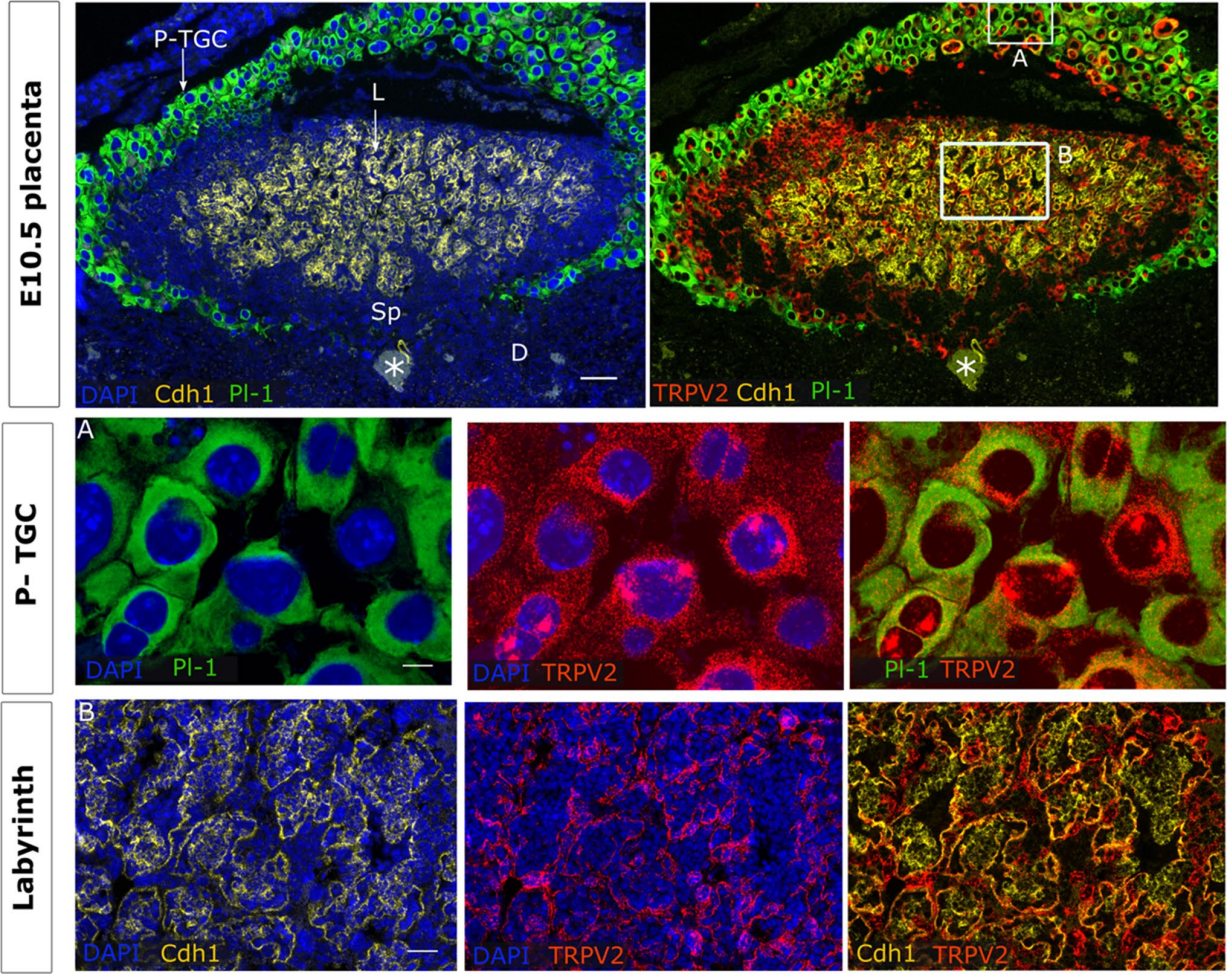
TRPV2 expression in premature placenta. mRNA in situ hybridisation of *Trpv2*, *Pl-1* as a marker for P-TGC, *Cdh1* as a marker for undifferentiated chorion and syncytiotrophoblasts-II cells. An overview of the E10.5 placenta is shown in the upper panels (scale bare = 200 µm). Magnifications of inserts A and B are presented below (scale bar = 25 and 50 µm). DAPI was used for nuclear staining. *P-TGC* parietal trophoblast giant cells, *L* labyrinth, *Sp* spongiotrophoblast, *D* decidua, *Pl-1* placental lactogen-1, *Cdh1* E-cadherin, *maternal artery

In E10.5 placentas, the premature labyrinth is distinguishable from the spongiotrophoblast layer by the presence of E-cadherin (*Cdh1*) positive clusters of undifferentiated cells. This premature placenta is surrounded by invasive Placental lactogen-1 (Pl-1) positive TGCs that initiate early embryo implantation. *Trpv2* was expressed in all PL1^+^ TGCs, in which signals were also observed in the nucleus, suggesting active transcription (Fig. 2, insert A). Moreover, *Trpv2* mRNA was present in the premature labyrinth, mainly in *Cdh1*^*−*^ cells. Co-expression with *Cdh1* was confined to cells outside cell clusters, suggesting expression in *Cdh1*^+^ syncytium trophoblasts-II cells [38] (Fig. 2, insert B).

**Fig. 3:**
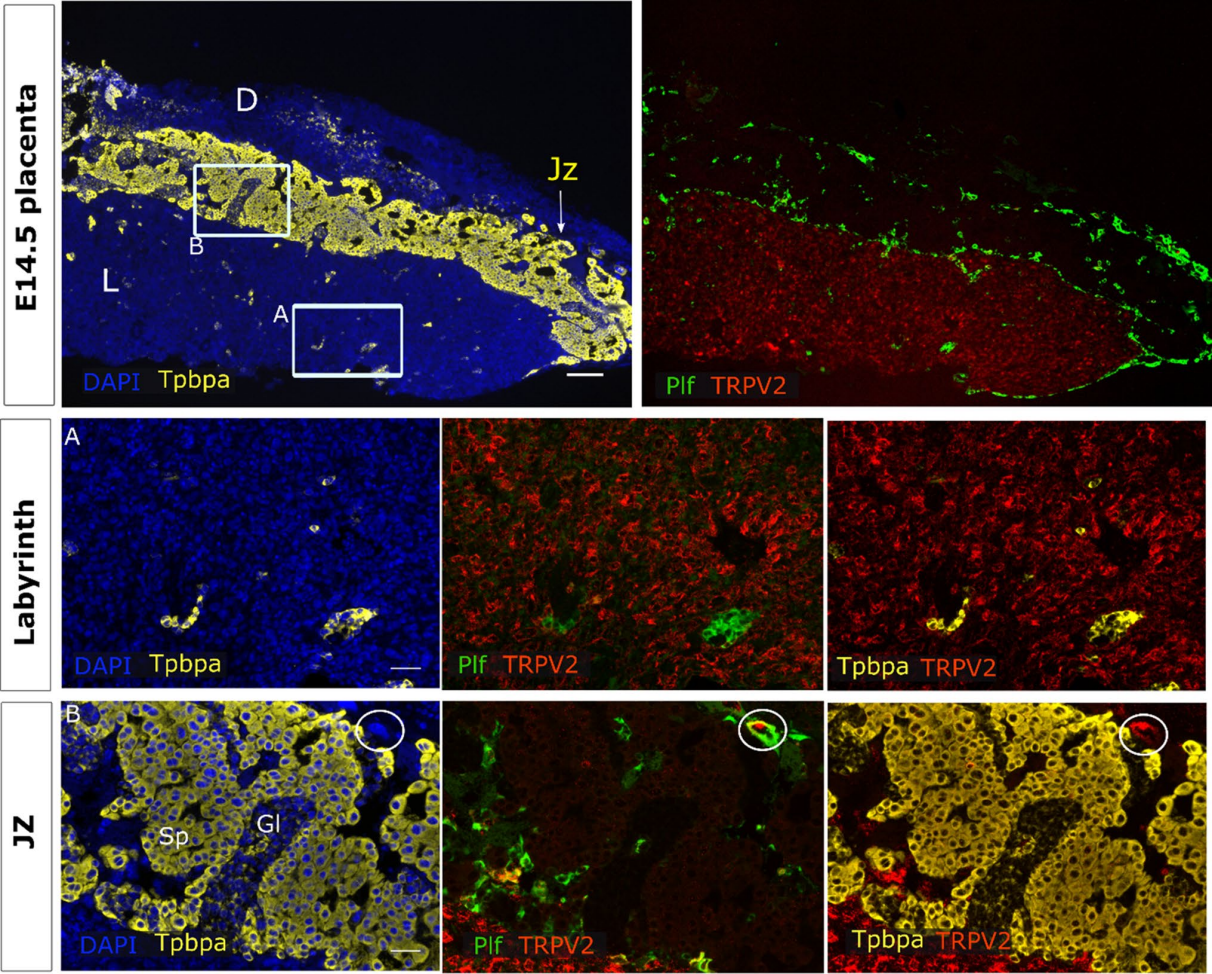
*Trpv2* expression in mature placenta. mRNA in situ hybridisation of *Trpv2*, *Tpbpa* as a marker for the junctional zone and *Plf* as a marker for certain TGCs. An overview of the E14.5 placenta is shown in the upper panels (scale bare = 200 µm). Magnifications of inserts A (Jz) and B (labyrinth) are presented below (scale bar = 50 µm). DAPI was used for nuclear staining. In A, a *Tpbpa*^−^/*Plf*^+^ TGC of the junctional zone is circled. *L* labyrinth, *Jz* junctional zone, *D* decidua, *Tpbpa* trophoblast-specific protein alpha, *Plf *proliferin

In line herewith, *Trpv2* mRNA levels remained detected in the labyrinth zone of the functional placenta (E14.5) (Fig. 3, insert A). *Tpbpa*^+^ spongiotrophoblasts and glycogen cells of the junctional zone showed almost no *Trpv2* expression. A positive signal was observed in *Tpbpa*^−^ TGC, which were often *Proliferin*^+^ (Plf) (Fig. 3, insert B). Strikingly, labyrinthine *Trpv2* expression was nearly absent at E18.5 (Supplementary Fig. 3, insert A), while expression in *Tpbpa*^−^/*Plf*^+^ TGCs remained. Moreover, very subtle expression in spongiotrophoblasts cells of the junctional zone was observed at E18.5, but not at E14.5 (Supplementary Fig. 3, insert B, arrowheads). Note that the expression in the endometrial stroma was used as a positive control (Supplementary Fig. 4a).

### TRPV6 was confined to the intraplacental yolk sac

**Fig. 4:**
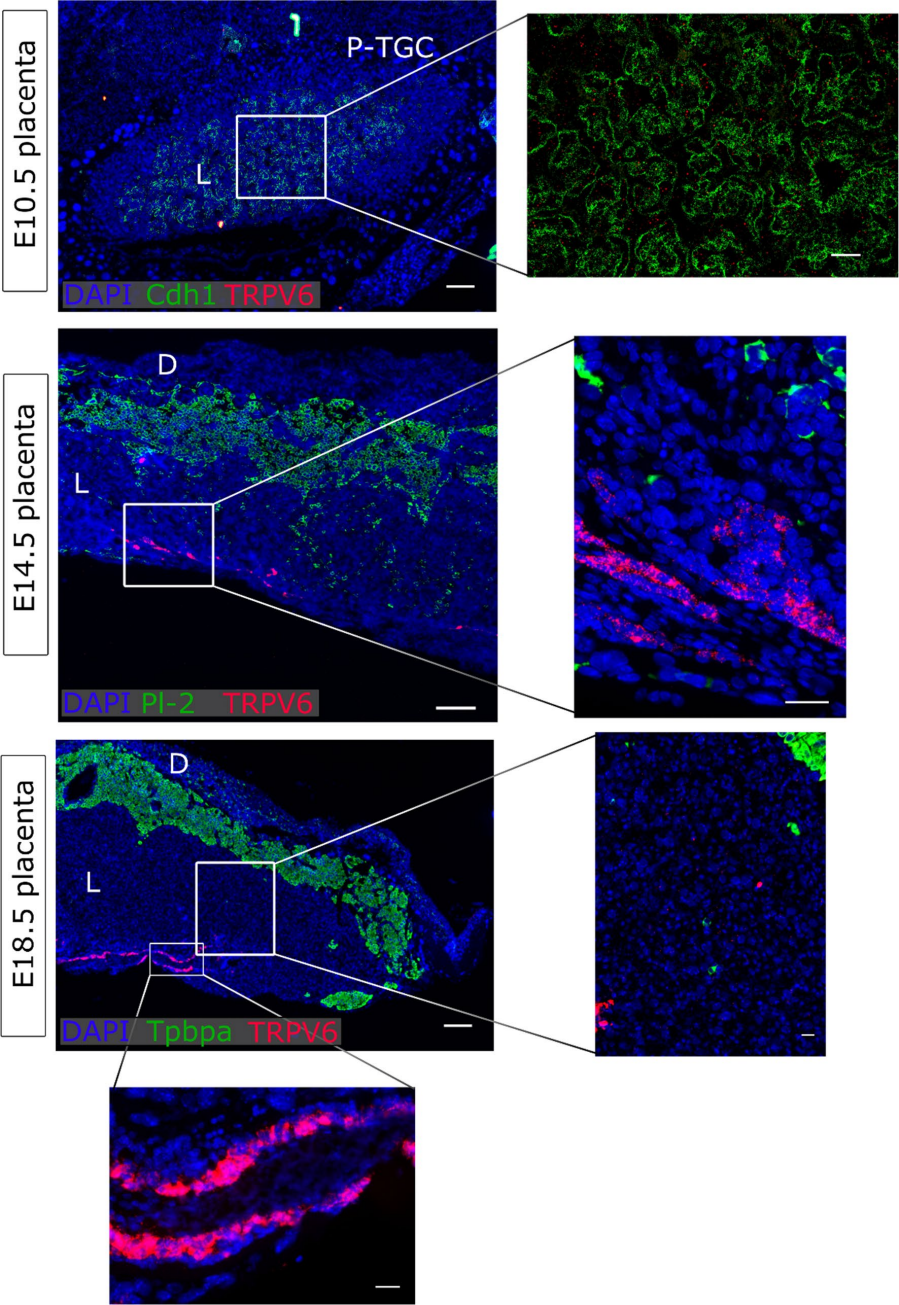
*Trpv6* expression in placenta. mRNA in situ hybridisation of *Trpv6* at E10.5, E14.5, and E18.5. At E10.5, and *Cdh1* for undifferentiated chorion and syncytiotrophoblasts-II cells. At E14.5, *Pl-2* was a marker for spongiotrophoblasts and secondary TGCs. At E18.5, *Tpbpa* was a marker for the junctional zone. An overview of the placentas is shown on the left (scale bare = 200 µm). Magnifications of inserts are presented right (scale bar = 50 µm at E10.5, and 25 µm at E14.5 and E18.5. DAPI was used for nuclear staining. *P-TGC* parietal trophoblast giant cells, *L* labyrinth, *D* decidua, *Cdh1* E-cadherin, *Tpbpa* trophoblast-specific protein alpha, *Pl-2* placental lactogen 2

At E10.5, only few *Trpv6* mRNA signals were observed in the labyrinth, and these did not show an obvious overlap with *Cdh1*^+^ cells of the chorion. In line, *Trpv6* signals were very weak in the junctional zone and in the labyrinth of mature placentas at E14.5 or E18.5. However, a very prominent expression was detected in the intraplacental yolk sac at both stages (Fig. 4). The presence of *Trpv6* mRNA signals in *Cdh1*^+^ epithelium of the endometrium served as a positive control (Supplementary Fig. 4d).

### TRPV4 and TRPM4 were detected in the maternal decidua

**Fig. 5:**
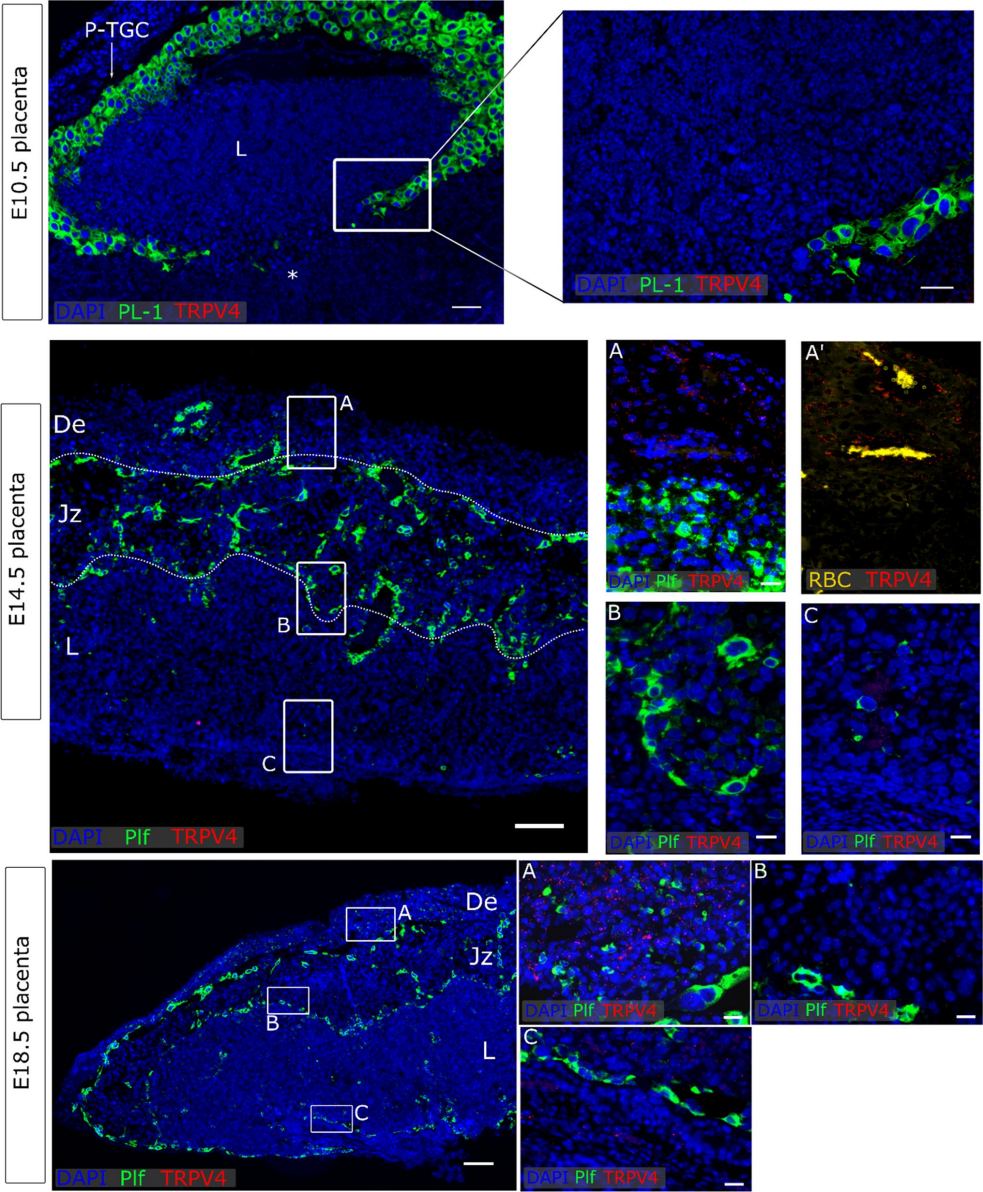
*Trpv4* expression in placenta. mRNA in situ hybridisation of *Trpv4*, *Pl-1* as a marker for the primary TGC, *Plf* as a marker for secondary TGCs. An overview of the E10.5 placenta is shown in the upper panels (scale bare = 200 µm) and magnifications of insert (scale bar = 100 µm). An overview of E14.5 placenta is shown in the middle panel (scale bar = 200 µm) and magnifications of inserts A (decidua), B (Jz) and C (labyrinth) (scale bar = 25 µm). A’ represent the same image as A in which RBC were shown. An overview of E18.5 placenta is shown in the lower panel (scale bar = 200 µm) and magnifications of inserts A (decidua), B (Jz) and C (labyrinth) (scale bar = 25 µm). DAPI was used for nuclear staining. *P-TGC* parietal trophoblast giant cells, *L* labyrinth, *Jz* junctional zone, *De* decidua, *Tpbpa* trophoblast-specific protein alpha, *Plf* proliferin, *RBC* red blood cells

No *Trpv4* could be detected in premature placentas at E10.5. Moreover, no *Trpv4* mRNA expression was noticed in neither the junctional zone nor the labyrinth of the mature placenta. However, diffuse expression was noticeable in the maternal decidua from both E14.5 and E18.5. Note that *Trpv4* mRNA in the decidua was rather low, and was detected in cells surrounding maternal arteries (Fig. 5). As a positive control, *Trpv4* was abundantly expressed in *Cdh1*^−^ vascular endothelial cells and to a lesser extent the *Cdh1*^+^ epithelium as well (Supplementary Fig. 4b). Additionally, *Trpv4* was strongly expressed in bladder urothelium (Supplementary Fig. 4c), as recently reviewed [39].

**Fig. 6:**
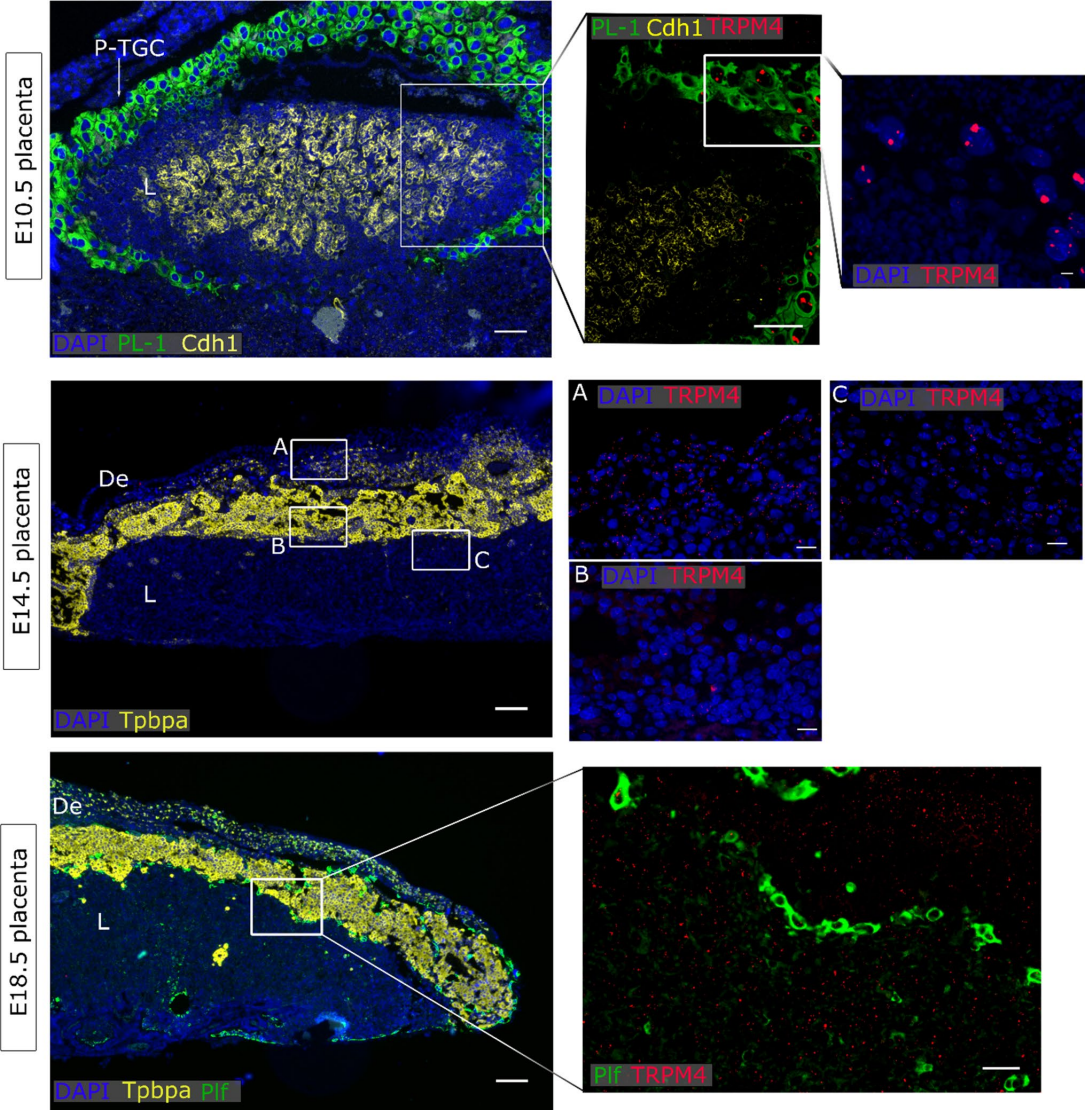
*Trpm4* expression in placenta. mRNA in situ hybridisation of *Trpm4*, *PL-1* as a marker for the primary TGC, *Cdh1* as a marker for undifferentiated chorion and syncytiotrophoblasts-II cells, *Tpbpa* as a marker for junctional zone cells, *Plf* as a marker for secondary TGCs. An overview of the E10.5 placenta is shown in the upper panels (scale bare = 200 µm) and magnifications of insert (scale bar = 100 µm and 25 µm). An overview of E14.5 placenta is shown in the middle panel (scale bar = 200 µm) and magnifications of inserts A (decidua), B (Jz) and C (labyrinth) (scale bar = 25 µm). An overview of E18.5 placenta is shown in the lower panel (scale bar = 200 µm) and magnifications of inserts (scale bar = 100 µm). DAPI was used for nuclear staining. *L* labyrinth, *Jz* junctional zone, *De* decidua, *Tpbpa* trophoblast-specific protein alpha, *Prl* proliferin,

In general, very low *Trpm4* mRNA levels were observed in the E10.5 placenta, although some spots were localized in the nucleus of some P-TGC, suggesting active transcription. In addition, *Trpm4* was diffusely expressed in the mature placenta at E14.5 and E18.5. Specifically, more staining was observed in the decidua at E14.5 (Fig. 6). Murine intestines were used as a positive control for *Trpm4* expression (Supplementary Fig. 4e).

In summary, these findings indicated a strong expression for *Trpv2* in trophoblast cells, while *Trpv4*, *Trpm4* were confined to the maternal decidua. *Trpv6* expression was specifically identified in the intraplacental yolk sac. Expression studies on separate placental layers confirmed the observations of FISH experiments as *Trpv2* was ~ sixfold higher expressed in the labyrinth compared to the decidua, whereas *Trpv4* and *Trpm4* were the highest in the maternal decidua. *Trpv6* expression was low, with ~ threefold higher expression in the labyrinth, in which the intraplacental yolk sac is contained. *Trpm6* and *Desmin* were used as controls for their specific expression in the labyrinth and decidua, respectively (Supplementary Fig. 2b, c; Table 2).

**Table 2:** Overview of TRP channel expression in placental development

	E10.5	E14.5	E18.5
Trpv2			
Location	Labyrinth Junctional zone	Labyrinth Junctional zone	Labyrinth Junctional zone
Cell type	Differentiated cells P-TGC	SynT-II, S-TGC Tpbpa^−^ TGC	SynT-II, S-TGC Tpbpa^−^ TGC
Expression	High High	High High	Low High
Trpv4			
Location		Decidua	Decidua
Cell type		Nd	Nd
Expression		Low	Low
Trpv6			
Location		Labyrinth	Labyrinth
Cell type		IPYS	IPYS
Expression		High	High
Trpm4			
Location	Junctional zone	Decidua	Whole placenta
Cell type	P-TGC (nucleus)	Nd.	Nd.
Expression	Low	Low	Low
Trpm6			
Location	Nd.	Labyrinth*	Labyrinth*
Cell type	Nd.	SynT-I*	SynT-I*
Expression	Nd.	High*	High*
Trpm7			
Location	Whole placenta	Whole placenta	Whole placenta
Cell type	Nd.	Nd.	Nd.
Expression	High	High	High

Empty boxes: no expression detected

*Nd.* specific expression not defined

*Data adopted from literature [35, 36]

### Functional expression pattern of TRP channels in primary mouse trophoblast cells

**Fig. 7:**
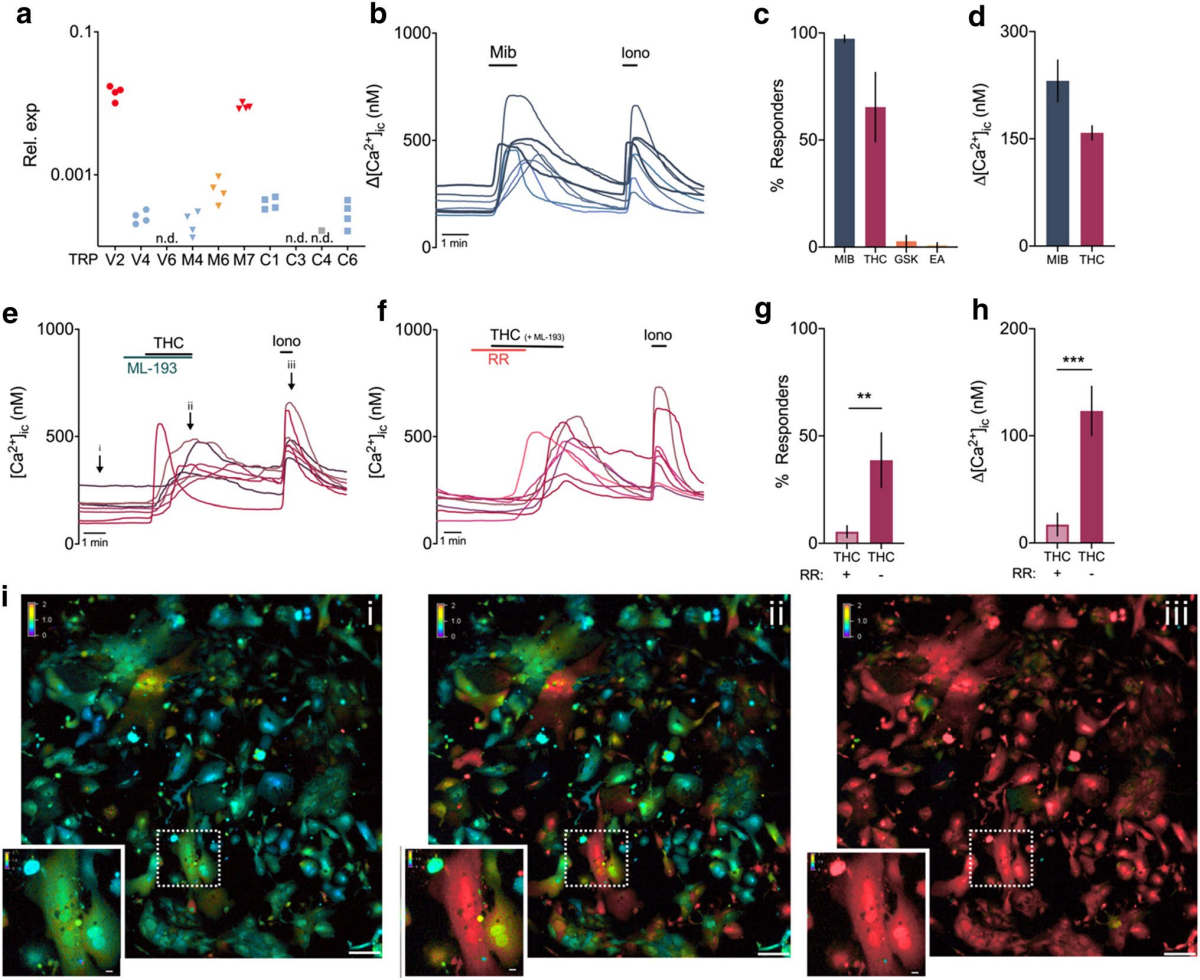
Functional expression of TRPV2 and TRPM7 in primary trophoblast cells at E14.5. **a** Relative expression of TRP channels that were above detection level in whole placental tissues, relative to the geometric mean of housekeeping genes *Gapdh* and *Actb*. Colours indicate expression level; red = high, orange = moderate, blue = low, and n.d. = below detection level. *N* = 4 cultures. **b** Example traces of Mibefradil (Mib, 200 µm)—induced intracellular calcium changes ([Ca^2+^]_i_), with each line representing a cell. **c** Percentage of responding trophoblast cells to activators (MIB: 1302 of 1336 cells; THC: 956 of 1470 cells; GSK 43 of 1475 cells; EA: 11 of 990 cells). **d** Amplitude of intracellular calcium increase in responding cells, represented as the difference between the peak value and the baseline value. **e** Example traces of Δ^9^-tetrahydrocannabinol (THC, 50 µm)—induced intracellular calcium changes, that could be blocked by the nonspecific TRPV inhibitor Ruthenium Red (RR, 2 µm) (**f**). ML-193 = specific GPR55 (cannabinoid receptor 3) blocker and was coapplied with THC. **g** Percentage of responders to THC in the presence (43 of 889 cells responding) and subsequent absence of RR (717 of 889 cells responding). **h** Amplitude of intracellular calcium increase of THC-responding cells during and after co-application with RR. Data are presented as mean ± SEM. Statistically significant changes were assessed with paired *T* test. *p < 0.05, ***p < 0.001. Ionomycin (Ion, 2 µm) was added at the end of every experiment as a positive control. *N* = minimum six experiments from minimum three independent cultures. (i) Representative colour-coded Fura-2 [Ca^2+^]_i_ ratio images during measurement of primary trophoblast cells indicated in graph **e**: basal situation (i), after THC application (ii), and after application of ionomycin (iii). Pseudo-colour ratio images were obtained using Nikon software. Scalebar = 100 µm. Insert shows multinucleated cell responsive to THC, scale bar = 10 µm

First, primary trophoblast cells were isolated and cultured at E14.5 from C57BL6/J mice. Using this method, cytokeratin-positive trophoblasts of both junctional and labyrinth zone were recovered, as shown by the expression of *Tpbpa* (marker of the junctional zone marker), *Hand1* (general TGC marker), *Slc16a1* and *Slc16a3* (marker of differentiated SynT-I and SynT-II, respectively), and *Ctsq* (marker of S-TGC) (Supplementary Fig. 5a–c). Moreover, specific morphology of trophoblast cells, such as giant cells or multinucleated cells, could be identified (Supplementary Fig. 5b). Nevertheless, some vimentin-positive mesenchymal cells could be identified, as well as low mRNA expression of *Pecam* (marker of endothelial cells), suggesting minor contamination of decidual or endothelial cells. The non-trophoblast cells were remarkably smaller in size and this was taken into account during functional experiments. Next, qRT-PCR was performed on the primary trophoblast cultures. Interestingly, the mRNA expression pattern was slightly different to the whole tissue expression. *Trpv2* and *Trpm6* were still considered high and moderately expressed compared to *Trpm7*, respectively. However, the expression of *Trpv4*, *Trpv6*, and *Trpm4* was low (Table 1; Fig. 7a). These findings corroborated the observations from the FISH experiments showing a lack of these channels in trophoblasts. Finally, to evaluate whether detection of mRNA resulted in a functional ion channel, calcium microfluorimetry was performed on primary trophoblast cells using specific TRP channel pharmacology. Only TRP channels that showed trophoblast expression in previous experiments and have commercially available agonists were considered.

First, functional activity of TRPM7 in trophoblast cells was assessed by stimulation with 200 µm mibefradil [30] and elicited a robust calcium influx (ΔCa^2+^ = 231 ± 23 nM) in 97 ± 2% of cells (Fig. 7b–d).

The functional expression of TRPV2 was evaluated by the application of 50 µm Δ9-tetrahydrocannabinol (THC), a known TRPV2 agonist [25]. Stimulation of trophoblasts by THC produced a rapid and reversible Ca^2+^ influx (ΔCa^2+^ = 158 ± 10 nM) in 65 ± 16% of the cells (Fig. 7c–e, i). Co-application of ML-193, the cannabinoid receptor 3 (GPR55) antagonist [40], was performed to exclude a significant contribution of this receptor to the THC-induced response. In the majority of the trophoblasts, the responses to THC stimulation were prevented by the nonspecific inhibitor of TRPV channels, ruthenium red (RR, 2 µm) [26, 27] as only 5 ± 3% responders were observed during co-application of RR and THC (Fig. 7f, g). Moreover, the calcium amplitude upon THC application was significantly lower during co-application of RR, (ΔCa^2+^ = 17 ± 11 nM compared to 123 ± 23 nM when RR was omitted) (Fig. 7h). Finally, THC-induced calcium influx was absent when calcium was omitted from the extracellular solution (ΔCa^2+^ = 15 ± 8 nM compared to 189 ± 13 nM in the presence of extracellular calcium) (Supplementary Fig. 6a). Application of the vehicle only did not induce calcium influx (Supplementary Fig. 6b).

In line with tissue expression, decreased TRPV2 expression was observed in E18.5 primary cultures (Supplementary Fig. 6d). This was translated in decreased functionality of TRPV2 as reduced amount of THC responders (38 ± 14%), and reduced THC-induced calcium amplitudes (ΔCa^2+^ = 135 ± 7 nM) (Supplementary Fig. 6e, f). Collectively, these results showed the responsiveness of trophoblasts towards stimulation by the TRPV2 agonist, THC.

The functionality of TRPV4 was evaluated by stimulation of trophoblasts with GSK016790A, a potent and selective activator [28]. At a concentration of 10 and 20 nM GSK016790A, a Ca^2+^ influx was detected in less than 3% of the cells, suggesting very low functional expression of TRPV4 in primary trophoblast cells (Fig. 7c; Supplementary Fig. 6c).

Next, stimulation of trophoblasts with Engelerin-A (250 nM), a specific activator of channels containing TRPC4 and TRPC5 with or without TRPC1, resulted in a significant rise in the intracellular calcium concentration in less than 1% of cells (Fig. 7c). These findings indicate the absence of functional TRPC1/4/5 channels in trophoblast cells and are in line with mRNA expression experiments.

All together, these data suggest that, in addition to its robust mRNA expression, TRPV2 and TRPM7 are functionally expressed in primary trophoblast cells.

### Molecular and functional expression of TRP channels during trophoblast stem cell differentiation

**Fig. 8:**
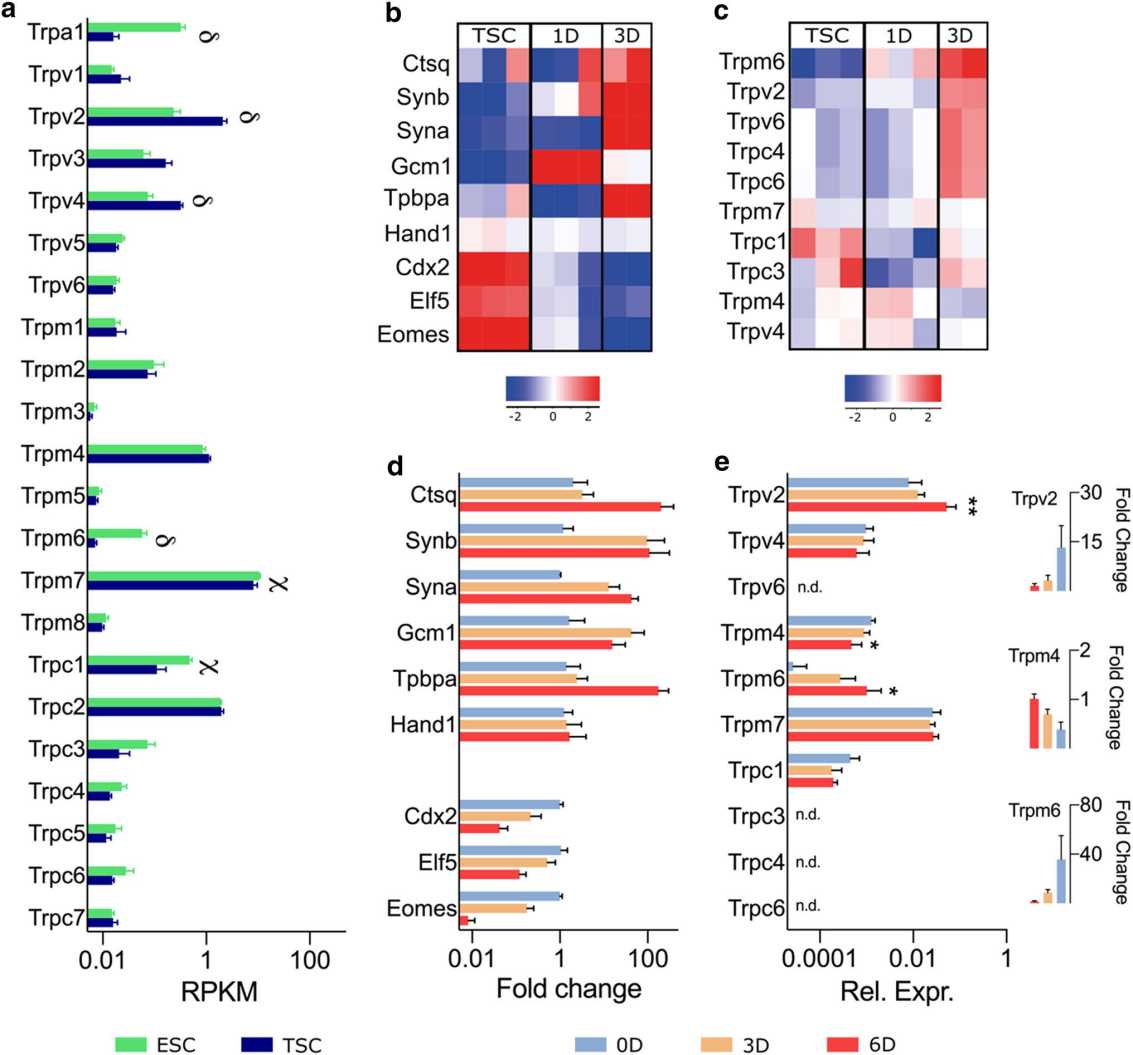
TRP channel expression in embryonic and trophoblast stem cells. **a** Expression of TRP channels in mouse ESC and TSC, shown as mean ± SEM. (*n* = 5). Differential expression was calculated using DESeq2 and adjusted for multiple testing correction using the Benjamini–Hochberg method. *γ*: *p* < 0.001, *δ*: *p* < 0.0001. Heat map of mean row-centered log2 RPKM during in vitro differentiation of mTSC as stem cells conditions, 1 day differentiation and 3 days differentiation, of marker genes (**b**) and TRP channels (**c**). Validation of gene expression with qRT-PCR in stem cell conditions (0D), 3 days and 6 days of differentiation (*n* = 4) of marker genes (fold change compared to 0D) (**d**) and TRP channels as relative expression and fold change of significantly changed genes (**e**). Data shown as mean ± SEM. Significant differences in mRNA expression were assessed with one-ANOVA followed by Dunnett’s multiple comparison test compared to 0D, using DeltaCT values; *p < 0.05, **p < 0.01. *RPKM* reads per kilobase of transcript, per million mapped reads, *ESC* embryonic stem cells, *TSC* trophoblast stem cells, *nd* not detected

At the stage of blastocyst (E4.5), two different cell populations emerge to form the trophectoderm and the inner cell mass establishing the trophoblast and embryonic cell lineages, respectively. Embryonic stem cells (ESC) have the ability to differentiate into all cell types of the embryo, whereas trophoblast stem cells (TSC) can differentiate into all the cell populations of the developing placenta [3, 4]. To gain insight into the expression of TRP channels during early placentation, their expression was assessed in mouse TSC and compared to mouse ESC using RNA-seq data that was retrieved from publicly available data sets [22, 23] (Fig. 8). As expected, *Trpm7* was abundantly expressed in both cell types, but significantly higher in mESC. The expression levels of *Trpv2* and *Trpv4* were significantly higher (2.7- and 1.6-fold, respectively) in mTSC compared to mESCs, whereas *Trpa1*, *Trpc1*, and *Trpm6* were significantly lower (5.6-, 2.6-, and 5.7-fold, respectively) (Fig. 8a).

Mouse TSC exhibit the potential to differentiate to all cell types of the placenta in vitro by the omittion of bFGF and conditioned medium (CM). This was shown by the downregulation of stemness markers *Cdx2*, *Elf5*, and *Eomes*, and the upregulation of differentiation markers for junctional zone cells (*Tpbpa*), TGCs (*Hand1*), and labyrinth cells including S-TGC (*Ctsq*), SynT-I (*Syna*), and SynT-II (*Gcm1*, *Synb*) (Fig. 8b). Since our previous results suggested temporal differences in the expression of some TRP channels, the expression dynamics in mTSCs during in vitro trophoblast differentiation were evaluated using the RNA-seq dataset (Fig. 8c). These data showed higher expression of *Trpm6*, *Trpv2*, *Trpv6*, *Trpc4*, and *Trpc6* at day 3 of differentiation. Using qRT-PCR, the expression profile was further validated in mTSC in stem cell conditions and at 3 and 6 days of differentiation. Similar to the RNA-seq data, mTSC differentiation resulted in the upregulation of trophoblast cell markers (Fig. 8d). In line with previous results, *Trpv2* and *Trpm7* were abundantly expressed in mTSC during differentiation. More specifically, expression levels of *Trpv2* were significantly upregulated at day 6 of differentiation. The relative expression levels were low for *Trpv4*, *Trpm4*, *Trpm6*, and *Trpc1* compared to *Trpm7* and below detection limit for *Trpv6*, *Trpc3*, *Trpc4*, and *Trpc6* (Table 1; Fig. 8e). Although average *Trpm6* levels were low, a 50-fold change at day 6 of differentiation was observed, which is in line with observations in intact placental tissues (Fig. 1) and with its specific expression in SynT cells.

Finally, the functionality of TRPV2 and TRPM7 was assessed in mTSC in stem cell conditions (0D) and after 6 days of differentiation (6D).

**Fig. 9:**
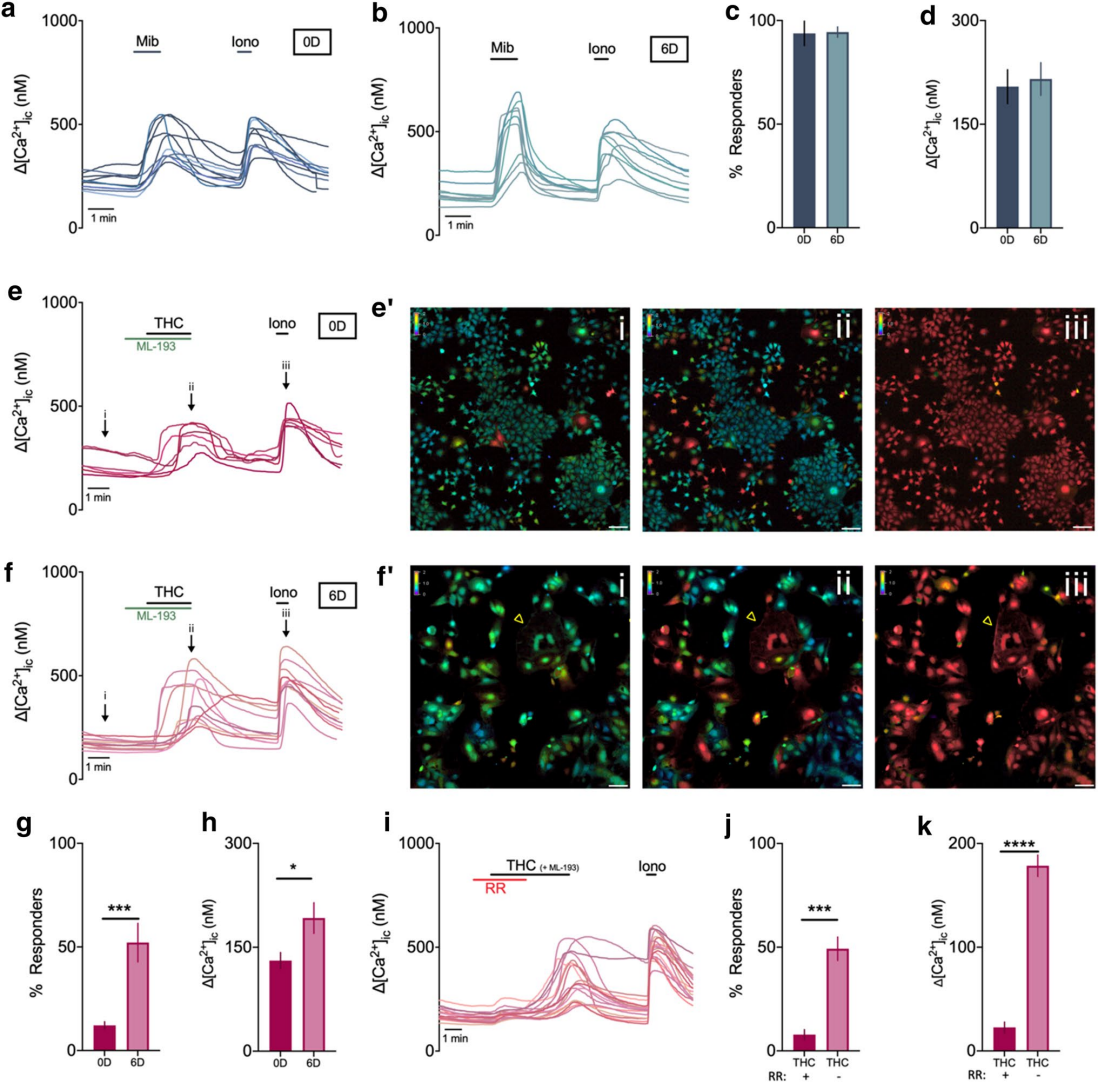
Functional expression of TRPV2 and TRPM7 in mTSC differentiation. Example traces of Mibefradil (Mib, 200 µm)—induced intracellular calcium changes ([Ca^2+^]_i_), with each line representing a cell, at 0D (**a**) and 6D of differentiation (**b**). **c** Percentage of responding mTSC to mibefradil at 0D (1709 of 1850 cells) and 6D (338 of 409 cells). **d** Amplitude of intracellular calcium increase in responding cells, represented as the difference between the peak value and the baseline value. *N* = 4 from two mTSC differentiations. Example traces of Δ^9^-tetrahydrocannabinol (THC, 50 µm)—induced intracellular calcium changes at 0D (**e**) and 6D (**f**) of differentiation. Representative colour-coded Fura-2 [Ca^2+^]_i_ ratio images of mTSC at 0D (**e′**) and 6D (**f′**) of differentiation at baseline (i), during application of THC (ii) and during application of ionomycin (iii), as indicated on graphs e and f. **g** Percentage of responding mTSC to THC at 0D (566 of 4511 cells) and 6D (480 of 864 cells); Fisher’s exact test. **h** Amplitude of intracellular calcium increase in responding cells; non-parametric Mann–Whitney test. *N* = 8–9 experiments from three mTSC differentiations. (i) Example traces of THC-induced calcium changes at 6D that could be blocked by the nonspecific TRPV inhibitor Ruthenium Red (RR, 2 µm). **g** Percentage of responders to THC during co-application with RR (63 of 803 cells responding) and after omission (417 of 803 cells). **h** Amplitude of intracellular calcium increase of THC-responding cells during and after co-application with RR. *N* = 6 from three mTSC differentiations. Paired *T* test. Data are presented as mean ± SEM. *p < 0.05; ***p < 0.001, ****p < 0.0001. Ionomycin (Ion, 2 µm) was added at the end of every experiment as a positive control. ML-193 = GPF55 antagonist

Stimulation with 200 µm Mibefradil induced a calcium influx (ΔCa^2+^ = 204 ± 24 nM) in the majority of mTSC (94 ± 6%) at 0D (Fig. 9a, c, d), and was not significantly altered by differentiation (ΔCa^2+^ = 215 ± 23 nM in 94.4 ± 2.5% of cells) (Fig. 9b–d).

In stem cell conditions, THC-induced a significant calcium influx (ΔCa^2+^ = 130 ± 12 nM) in 12 ± 2% of cells (Fig. 9e, g, h). In line with significantly increased *Trpv2* mRNA levels after 6 days of differentiation, TRPV2 functionality was increased as well. As such, THC application induced a response in significantly increased number of cells (52 ± 9%) and was associated with significantly higher calcium amplitude (ΔCa^2+^ = 192 ± 22 nM) (Fig. 9f–h). Representative images obtained during calcium microfluorimetry demonstrated the specific morphology of colonies of small cells at 0D and large differentiated cells at 6D of differentiation, including multinucleated cells (arrowhead) (Fig. 9e′, f′, respectively). The responses to THC stimulation were prevented by co-application of RR, as only 8 ± 2% responders were observed, which increased to 49 ± 6% responding cells upon omission of RR (Fig. 9i, j). In line herewith, the calcium amplitude of THC-responding cells was significantly lower during co-application of RR (ΔCa^2+^ = 23 ± 2 nM compared to 178 ± 10 nM when RR was omitted) (Fig. 9k).

Overall, these results indicate that the expression of TRP channels is a dynamical process across the mouse placental development that may be further investigated in detail using in vitro culture of mTSCs.

## Discussion

TRP channels excel in interpreting signals from their environment and conveying this information into a cellular response, often using calcium as a secondary messenger [6]. Moreover, they are non-selective cation channels involved in cellular transport of sodium and magnesium as well. In recent years, a role for TRP channels in invasion, migration, differentiation, hormone secretion, and angiogenesis has emerged [41]. For this reason, research focusing on TRP channels in pathogenesis where said processes are dysregulated, such as tumorigenesis, is becoming increasingly valued. Although these processes are all instrumental in proper placental development as well, a comprehensive understanding regarding TRP channels expression and their possible function in the placenta is missing. Here, *Trpv2*, *Trpv4*, *Trpv6*, *Trpm4*, *Trpm6*, and *Trpm7* were considered for further examination to assess specific spatial expression in the different placental layers. The outcome of this study confirmed the expression of TRPV6, TRPM6, and TRPM7 in the mouse placenta, and identified the prominent expression of TRPV2 in trophoblast cells, both on molecular as functional level.

### A role for TRP channels in cellular physiology

TRPV2, TRPV4, TRPM4, and TRPM7 have been described in several of physiological processes, and have been linked to increased invasiveness during tumorigenesis [42, 43]. Therefore, these channels might have a similar role in trophoblast invasion during placental development.

TRPV2 remains one of the most enigmatic TRP channels because its specific physiological role is largely unknown. Interestingly, TRPV2 knockout mice have a reduced birth weight and are more susceptible to perinatal lethality, a feature that was attributed to prenatal events rather than post-natal failure to thrive [18]. Thus, TRPV2 might have a potential role in embryonic or placental development. Here, we observed very abundant mRNA expression levels of TRPV2 in whole placental tissues, which decayed towards term. At E10.5 of gestation, very prominent expression was observed in *Pl-1*^+^ P-TGC. In contrast to other trophoblasts, these TGC differentiate from mural trophectoderm cells instead of polar trophectoderm. During early gestation, P-TGC are involved in embryonic implantation, as they invade the decidua and connect to the maternal vasculature to initiate maternal–fetal transport. They completely surround the invading conceptus by remodelling of the extracellular matrix, phagocytosis, and significant cell motility [44]. Interestingly, TRPV2 function has been described in cell migration and phagocytosis as well, hinting to a similar purpose in P-TGCs [45]. In addition, *Trpv2* was dispersedly expressed in the premature labyrinth, in both *Cdh1*^+^ *and Cdh1*^*−*^ cells. Cdh1 is an epithelial marker that is highly present in stem cells and thus in undifferentiated chorion. During differentiation, trophoblast stem cells will undergo epithelial-to-mesenchymal transition (EMT), thereby loosing Cdh1 expression. Interestingly, syncytiotrophoblasts layer II, but not layer I, cells do not require downregulation of Cdh1 during EMT [38, 46]. These findings suggest that TRPV2 is likely expressed in differentiated labyrinth cells, but not in undifferentiated stem cells. In line with these findings is the increasing expression of *Trpv2* during in vitro differentiation of mTSC towards differentiated trophoblasts. Not surprisingly, *Trpv2* was highly expressed in the mature labyrinth as well, as shown by FISH and qRT-PCR on separated placental layers. Interestingly, *Trpv2* and *Tpbpa* seemed to be mutually exclusive, suggesting that *Trpv2* is not expressed in spongiotrophoblasts or glycogen trophoblast cells. However, *Tpbpa*^−^/*Plf*^+^ cells were positive for *Trpv2*, suggesting expression in some Canal TGC, Channel TGC, and P-TGC, which are 50% Tpbpa^+^, but not Spiral Artery TGCs as they are 100% Tpbpa^+^ [47]. At term, a similar expression pattern was observed in TGCs, while labyrinthine *Trpv2* mRNA expression was drastically decreased. Furthermore, functional expression was evaluated using calcium microfluorimetry in primary trophoblast cultures. In line with the high mRNA levels in primary cultures, TRPV2 activity was detected in more than 60% of trophoblast. This THC-induced calcium influx was challenged with ML193 and the TRPV blocker Ruthenium Red. The former is a known blocker for Cannabinoid receptor 3 (GPR55), a protein that is activated by THC and is able to produce an increase in intracellular calcium levels [40]. TRPV2 activity was significantly reduced at E18.5, in line with decreased mRNA levels both in situ and in vitro. However, as primary trophobalsts cells might contain some mesenchymal contamination, functional experiments were validated using mTSC. In line with increased mRNA expression levels, TRPV2 functionality statistically increased after 6 days of differentiation compared to stem cell conditions. Therefore, our results provide evidence for a robust THC-induced calcium influx governed by TRPV2. Collectively, TRPV2 is highly abundant in the placenta and is functionally present in primary murine trophoblasts. The distinct expression profile indicates that TRPV2 might fulfill diverse functions, such as contributing to invasion or migration, phagocytosis or other, so far unknown functions.

TRPV4 expression in whole placental tissues was moderate and showed temporal changes during gestation. Spatial expression assessed with FISH revealed that *Trpv4* expression in the fetal placenta, e.g. the junctional zone and the labyrinth, was not detectable. Subtle expression was noticed in the maternal decidua at E14.5 and E18.5, including in cells lining uterine vessels. In addition, qRT-PCR on separated layers at E14.5 confirmed that *Trpv4* was mainly detected in the decidua. Expression of TRPV4 in vascular endothelial cells would be consistent with previously reported endothelial expression [48] and with the high expression observed in uterine endothelial cells (Supplementary Fig. 4c). During gestation, *Tpbpa*^+^/*Plf*^+^ TGC migrate into the decidua and remodel spiral arteries (Sp-TGC) into low resistant vessels, thereby replacing endothelial cells [47]. However, *Trpv4* positive cells within the decidua were *Plf*^*−*^ and thus refute a TGC character. Other trophoblast cells residing in the decidua are *Tpbpa*^+^ glycogen cells that migrate during mid-gestation from the junctional zone towards the decidua [49]. Although co-expression with *Tpbpa* was not performed, the absence of *Trpv4* in the entire junctional zone, including glycogen cells, implied that *Trpv4* positive cells are most likely not glycogen cells. In line with these findings, functional expression of TRPV4 was found in a very small percentage of primary trophoblast cells (< 3%). These results indicate that TRPV4 is not functionally present in trophoblast cells and that its expression in the placenta is most likely limited to cells of the decidua, endothelial cells, stromal cells [21, 50] or otherwise. In line herewith, *Trpv4* was poorly expressed in mTSC and did not show remarkable changes during in vitro differentiation. As such, TRPV4 will have a limited role in placental development, and is consistent with the absence a reproductive phenotype in TRPV4 KO mice [51, 52]. Nevertheless, a significant contribution of TRPV4 in uterine contraction preceding labour has been described [53–55].

Unlike other members of the TRP family, TRPM4 and TRPM5 represent calcium-activated, but calcium impermeable, monovalent cation channels [56]. TRPM4 expression is widespread in excitable and non-excitable cells throughout the body, with the highest levels in the intestines [57]. In whole placental tissues, *Trpm4* was moderately expressed, with limited alterations over time. Importantly, strain-dependent differences were observed in that the overall placental expression of *Trpm4* was higher in the mixed BL6/129S background. Although no subfertile phenotype is reported for TRPM4 knockout mice so far with either 129/Svj or C57BL/6J background, it is interesting to note that strain-specific cardiac phenotypes were observed [58–62]. While the entire placental *Trpm4* levels were above threshold, spatial FISH expression studies revealed very low expression in the placenta. Like *Trpv4*, also *Trpm4* was most abundantly expressed in the maternal decidua, which was confirmed by qRT-PCR on separated placental layers. In line with these findings, the expression of *Trpm4* in primary trophoblast cultures was no longer of significance and further functional characterization was deemed redundant. Additionally, *Trpm4* expression was low in mTSC and did not alter upon differentiation.

TRPM7 is the most abundantly and ubiquitously expressed TRP channel in the adult body. Together with TRPM6, it has both a channel and a kinase function, and thereby distinguish themselves from other TRP channels as chanzymes [63, 64]. TRPM7 is instrumental in magnesium homeostasis as it is highly permeable for magnesium, but is also inhibited by physiological concentrations of intracellular and extracellular magnesium [37]. In the current study, *Trpm7* was highly expressed in intact placental tissues and showed little variation during gestation. Moreover, *Trpm7* transcripts were observed in primary cultures and its functionality, assessed as mibefradil-induced calcium influx [30], was found in almost all cells. These findings are of no surprise, given the housekeeping function of TRPM7 in most cell types. Suzuki et al. previously reported the presence of TRPM7 homomers in E14.5 trophoblasts as well, identified as currents that were observed by the chelation of magnesium and that were inhibited by 2-APB [36]. In line herewith was the presence of TRPM7 in mTSC before and after differentiation. Interestingly, mTSC lacking TRPM7 failed to proliferate in vitro, unless additional Mg^2+^ was provided. [35]. Moreover, TRPM7-deficient cells were characterized by reduced cellular Mg^2+^ content, supporting a pivotal role of TRPM7 in cellular Mg^2^ uptake of mTSC. However, embryonic lethality that was caused by global disruption of TRPM7 could not be rescued by restoring its function in extraembryonic cells. These findings emphasize an essential role of TRPM7 during embryogenesis and organogenesis, while its functions seems redundant in placental development [65–67]. More specifically, it was shown that TRPM7 was essential only during the early stages of embryogenesis (E7-E9) [68]. As such, a crucial role for TRPM7 in the proliferation of mESC was demonstrated and could be attributed to the kinase function of the channel [69]. Taken together, TRPM7 is functionally expressed in all of the different cell populations of the mouse placenta, although its specific role remains elusive.

### A role for TRP channels in placental transport of cations

Although intracellular calcium is pivotal in normal cellular physiology, where it often seen as the executive power in processes as invasion and migration, calcium per se has to be transported to the developing fetus as well. Fetal blood levels are maintained hypercalcemic compared to maternal levels during late pregnancy, suggesting that calcium is in part actively transported across the placenta. Therefore, placental calcium transport must involve (1) calcium entry at the maternal side according to the concentration gradient, (2) intracellular transport of buffered calcium to prevent  a calcium response, and (3) active calcium extrusion at the fetal side.

TRPV6 channels are excellent candidates to govern the initial calcium influx. First, TRPV6 shows a high permeability for calcium (P_Ca_/P_Na_ = 100) [70]. Second, we and others [71] have demonstrated a tenfold increase in *Trpv6* expression in whole placental tissue towards term, in line with the increasing fetal demands for calcium ions. Indeed, 80% of the fetal calcium in human is accumulated in the third trimester alone. Similarly, about 12 mg calcium is transported in the remaining 5 days of gestation in rat, in contrast to less than 0.5 mg calcium in the first days [72]. Third, a similar role for TRPV6 in calcium transport has been described in renal, intestinal, and epididymal calcium uptake [73]. Remarkably, TRPV6 is more abundantly expressed in the placenta compared to intestines or kidney [74]. Finally, co-expression of TRPV6 and Calbindin D, a calcium binding protein that functions as an intracellular buffer, has been described in the human placenta [75–77]. Most maternal–fetal nutrient transfer occurs in the labyrinth, where the juxtaposition of maternal and fetal circulations allows for an optimal exchange. However, the site of active calcium transport in rodents is likely through the endoderm of the intraplacental yolk sac, shown by the localisation of ATP-dependent calcium transport and other calcitropic genes [78, 79]. As such, we and others [71], observed significant mRNA expression of *Trpv6* in the intraplacental yolk sac, while no mRNA expression could be observed in other regions of the placenta at E10.5, E14.5 or E18.5. These results were further confirmed by the absence of *Trpv6* expression in primary trophoblast cultures, and the very low expression in mTSC. Therefore, the exponential increase in TRPV6 expression during pregnancy is most likely caused by significantly increased expression in the intraplacental yolk sac, but not in trophoblast cells.

Collectively, our results are in line with previous reports and suggest that TRPV6 is mainly present in the yolk sac where it might play a prominent role in calcium transport for the developing fetus. Given that calcium must be actively transported against its gradient, it is not completely surprising that this transport does not occur in the labyrinth, in which the fetal and maternal circulations are separated by four different cell layers, i.e. the fetal endothelium, two syncytium trophoblast layers, and sinusoidal TGC. Indeed, the intraplacental yolk sac provides a more effective route as it resides between thin-walled fetal vessels and maternal blood spaces at the fetal pole of the placenta [79]. Interestingly, this tissue was shown to actively invaginate and expand in volume during the last 5 days of gestation, coinciding with the time frame of rapid calcium transfer [80]. Nevertheless, different expression patterns of TRPV6 have been described as well. As such, Lee et al. reported that placental mRNA expression peaked at E10 of gestation and was moderately expressed at E14 and E17. Moreover, TRPV6 protein expression was observed in the labyrinth, the junctional zone and fetal membranes [81]. More recently, TRPV5 and TRPV6 were assessed in the placenta by another group, describing expression of TRPV6 in the labyrinth and the junctional zone. In addition, they reported the strongest expression of TRPV6 in the junctional zone, while the decidua lacked TRPV6 [82]. Adding to this discord, Fecher-Trost et al., reported TRPV6 protein expression in the labyrinth and the decidua, but not in the junctional zone [83]. The current study thus identified important discrepancies in the TRPV6 expression pattern compared to previous studies. Here, FISH experiments were used to identify *Trpv6* transcripts. The specificity of the probe was further validated by very prominent expression in the uterine epithelium, in line with previous reports [84].

Interestingly, important differences with the human placenta can be observed. First, TRPV5 and TRPV6 are co-expressed in the human placenta and cooperate in calcium uptake. [75–77]. Our results indicate that *Trpv5* was not expressed in the mouse placenta. Second, TRPV6 was found in human cytotrophoblasts and increased during differentiation into syncytiotrophoblasts [85]. This is substantially different from the high expression in the intraplacental yolk sac and the absence in the syncytiotrophoblasts of the labyrinth. However, a plausible explanation is that the site of calcium transport in the human placenta is at the fetus-facing basement membrane of the syncytiotrophoblasts. Moreover, a corresponding structure of the intraplacental yolk sac in human or primates has not been described. Therefore, TRPV6 is likely to play a role in calcium transport in both human and mouse placentas, albeit via different transport routes. Supporting this idea is the decreased maternal–fetal calcium transport as a result of missense mutation in human TRPV6. Skeletal abnormalities presented at birth were resolved during the first few months of the neonates’ life, and raised, therefore, the possibility of insufficient maternal–fetal transport rather than a primary disease causing deficient calcium uptake [86, 87].

Though TRPV6 plays an instrumental role in calcium transport, placental transport of radioactive calcium in TRPV6 knockout mice was not completely abolished, as it was reduced by only 40% [71]. In this regard, TRPM6 was identified as an additional candidate to govern apical calcium entry [36]. Similar to TRPV6, we and others observed a strong increase in *Trpm6* transcripts in whole placental tissue towards term. Indeed, Suzuki et al. further identified that *Trpm6* mRNA and protein was confined to the labyrinth [36], whereas Chubanov et al. specified its expression to the visceral yolk sac endoderm and the extraembryonic chorion, more explicitly the SynT-I cells at E8.5 and syncytiotrophoblast at E14.5 [35]. Moreover, we observed a 50-fold increase of *Trpm6* during mTSC differentiation, which is in line with the upregulation of the SynT-I marker *SynA*. However, it has been shown that TRPM6 is essential for magnesium rather than calcium transport. Indeed, heterozygous deletion of TRPM6 in mice results in hypomagnesemia whereas serum calcium levels were unaffected. Interestingly, mice completely lacking TRPM6 are embryonically lethal mainly due to neural tube defects [88, 89]. Counterintuitively, *Trpm6* transcripts could not be identified in the neural tube, suggesting that *Trpm6* expression during development was mostly confined to the extraembryonic tissues [35]. Excitingly, restoring the function of TRPM6 in these extraembryonic tissues using an epiblast-driven knockout strategy was compatible with viable offspring. These findings provide strong evidence that extraembryonic TRPM6 is indispensable during development. In our study, we observed strain-specific differences in TRPM6 expression in that C57BL6/J placentas have lower TRPM6 levels. However, C57BL6/J have disproportionally larger junctional zone and, therefore, a reduced labyrinth density. As such, labyrinth markers such as *Dlc3*, *Gcm1*, and *SynA* were lower in C57Bl6 placenta compared to 129Sj [33]. Lower TRPM6 levels in C57BL6/J placentas might thus be a consequence of this decreased labyrinth proportion. These findings raise the questions whether the phenotype of TRPM6 knockout mice would be strain-specific, as shown for other placental phenotypes such as the EGFR [90, 91].

Previous studies have investigated TRPM6 expression and functionality in much more details. As such, [Mg^2+^]_i_- and [MgATP]_i_-sensitive divalent cation currents in mTSC were found to be carried by TRPM6/TRPM7 heteromers [35]. In this regard, the authors proved that association of TRPM6 with TRPM7 as functional heteromers mitigates the tightly controlled inhibition of TRPM7 homomers by cytosolic levels of [Mg^2+^]_i_ and [Mg·ATP]_I_. While this relieve will result in facilitated magnesium entry, it is probably not required for cell autonomous function as the lack of TRPM6 did not affect the self-renewal capacity of mTSC [35]. Moreover, Suzuki et al. reported TRPM6/TRPM7 currents at E18.5 but not at E14.5 of pregnancy. Collectively, TRPM6 is functionally present mTSC [35] and primary trophoblast cells [36] and is essential for magnesium transport, rather than calcium transport.

Recently, a dataset containing single nuclei RNA sequencing (snRNA-seq) at multiple stages of mouse embryonic development was made available [38], and was used to verify our obtained results (Supplementary Fig. 7a). In line with our findings, *Trpv2*, *Trpm6*, and *Trpm7* were abundantly expressed in specific cell clusters, whereas *Trpv4*, *Trpv6*, and *Trpm4* could only be observed in minority of nuclei (Supplementary Fig. 7b, c). Interestingly, *Trpv2* was confined to SynT-II and S-TGC, and their respective precursors (Supplementary Fig. 7c, d). *Trpm6* has been shown to be expressed in SynT-I cells at E8.5 and in syncytiotrophoblasts at E14.5 [35]. Indeed, the snRNA-seq data revealed that *Trpm6* expression in SynT-I cells increased during gestation and a similar, so far unidentified, pattern could be observed for SynT-II as well (Supplementary Fig. 7c, e). Our experiments failed to detect *Trpv6* in trophoblasts cells, although high expression was observed in the intraplacental yolk sac. Likewise, no *Trpv6* transcripts were detected in the snRNA-seq in trophoblast, but a high, and very specific pattern was observed when plotting all cell present in the maternal–fetal interface (Supplementary Fig. 7f). Interestingly, the authors of the snRNA-seq dataset [38] identified the nature of this cluster (cluster 15) as unclear. Together, this might suggest that *Trpv6* can be used as a marker to identify cells of the intraplacental yolk sac, and that Cluster 15 and 16 might thus represent the parietal and visceral yolk sac. Moreover, *Trpm6* was also reported to be expressed in the yolk sac, as observed here as well. Collectively, the findings of the present study could all be validated using the recently published snRNA-seq dataset.

In conclusion, the current study both confirmed the expression of TRPV6, TRPM6, and identified the high expression of TRPV2 and TRPM7 in the mouse placenta. These findings suggest that aberrant TRP channel function might results in poor placental functioning. Together, our findings indicate that investigating TRP channel function during placental development holds great potential to further understand placental pathologies. Finally, the strong similarity in TRP channel expression between primary trophoblasts and during mTSC differentiation endorse the use of mTSC to study TRP channels in placental development.

### Supplementary Information

Below is the link to the electronic supplementary material.

Supplementary file1 (DOCX 7679 kb)
