# Supplementary material for: Mapping the expression of transient receptor potential channels across murine placental development
Source: Cell Mol Life Sci. 2021 Apr 21;78(11):4993–5014. doi: 10.1007/s00018-021-03837-3 (PMC8233283; doi:10.1007/s00018-021-03837-3)
Supplement: Supplementary file 1 — Supplementary file1 (DOCX 7679 kb) [file 18_2021_3837_MOESM1_ESM.docx]

# SUPPLEMENTARY INFORMATION


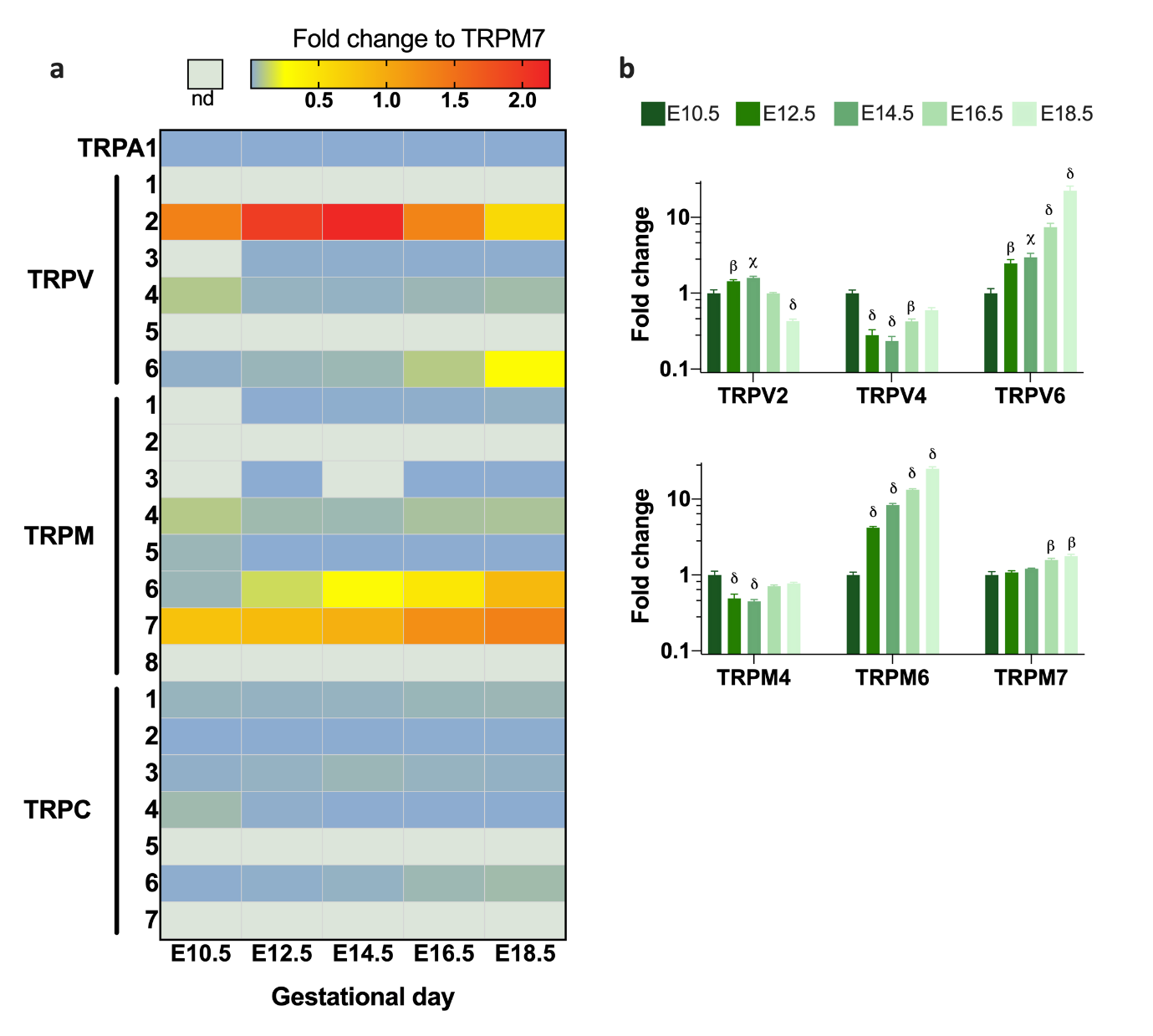


**Supplementary Fig. 1 Quantitative RT-PCR showing TRP channel expression in placentas from mixed BL6/129S mice through gestation**

(a) Heat map of mRNA levels of TRP channels relatively quantified to the geometric mean of housekeeping genes *Actb* and *Gapdh* and then normalized to *Trpm7* expression. nd = non detectable (b) Normalized fold change of expressed TRP channel, compared to E10.5, shown as mean ± SEM. Significant differences in mRNA expression were assessed with One-way ANOVA followed by Dunnett’s multiple comparisons test compared to E10.5, using DeltaCT values. 𝛂: p < 0.05, β: p < 0.01, 𝜸: p < 0.001, δ: p < 0.0001. E = embryonic day. n = 4 placentas from 2 different litters.


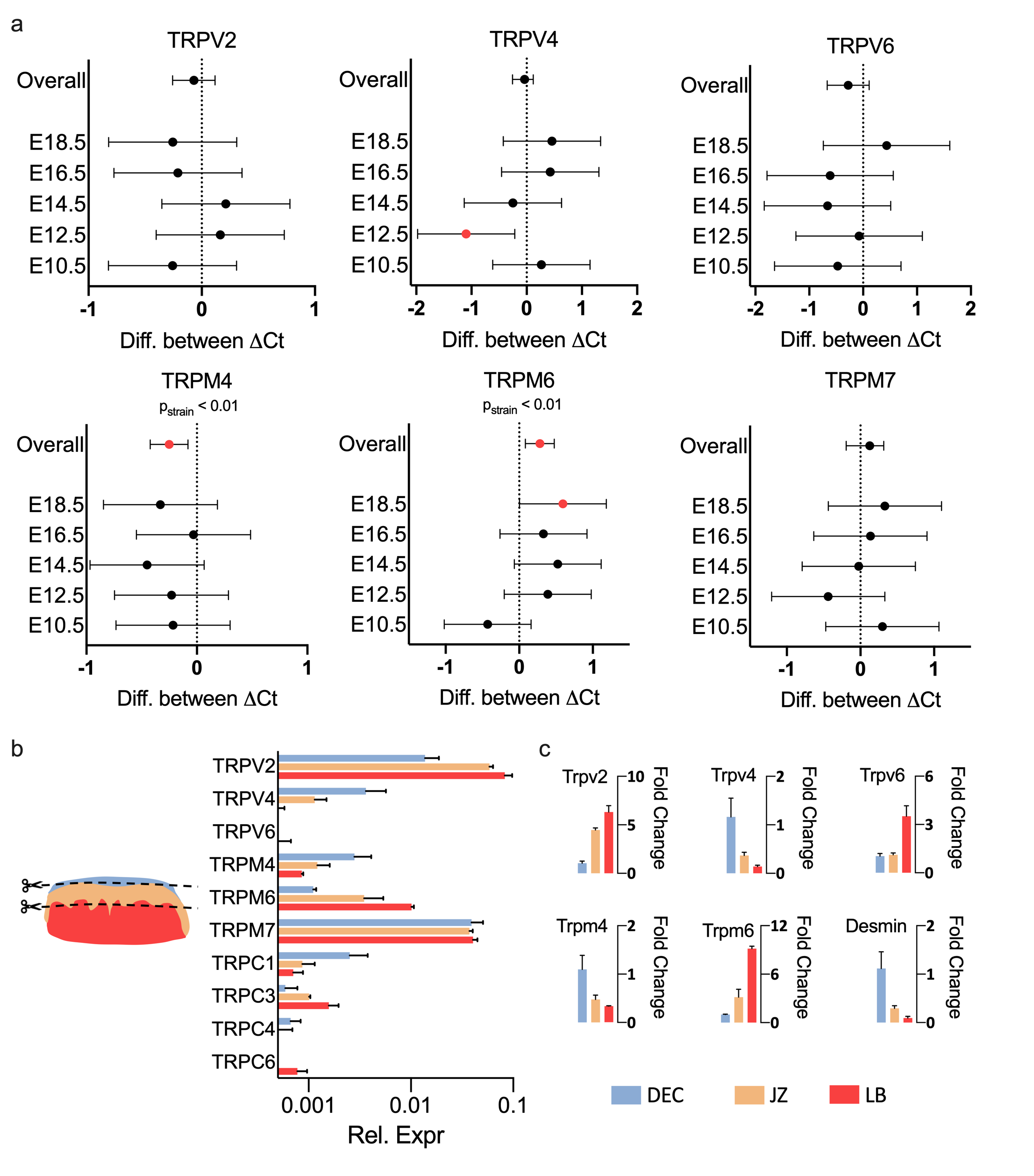


**Supplementary Fig. 2 Strain specific and layer specific expression patterns**

(a) Confidence interval (CI) of the mean difference between ΔCt values of C57BL6 and mixed B6/129S mice, shown as the overall difference independent of gestational day (p_strain_) and posthoc analysis comparing different gestational days. Positive values indicate higher expression in the mixed BL6/129S6 mice, negative values indicate higher expression in C57BL6 mice. Statistical differences were calculated with Two-way ANOVA using ΔCt values, followed by the post hoc Bonferroni’s multiple comparisons test. (b) Relative expression of TRP channel in mechanically separated placental layers: maternal decidua (DEC), the junctional zone (JZ) and the labyrinth zone (LB). (c) Normalized fold change of TRP channel assessed with FISH, compared to the decidua, shown as mean ± SEM. Trpm6 (shown to specific for the labyrinth [35,36]) and Desmin (expressed in the decidua) as positive controls. N=3 from 3 litters.

**
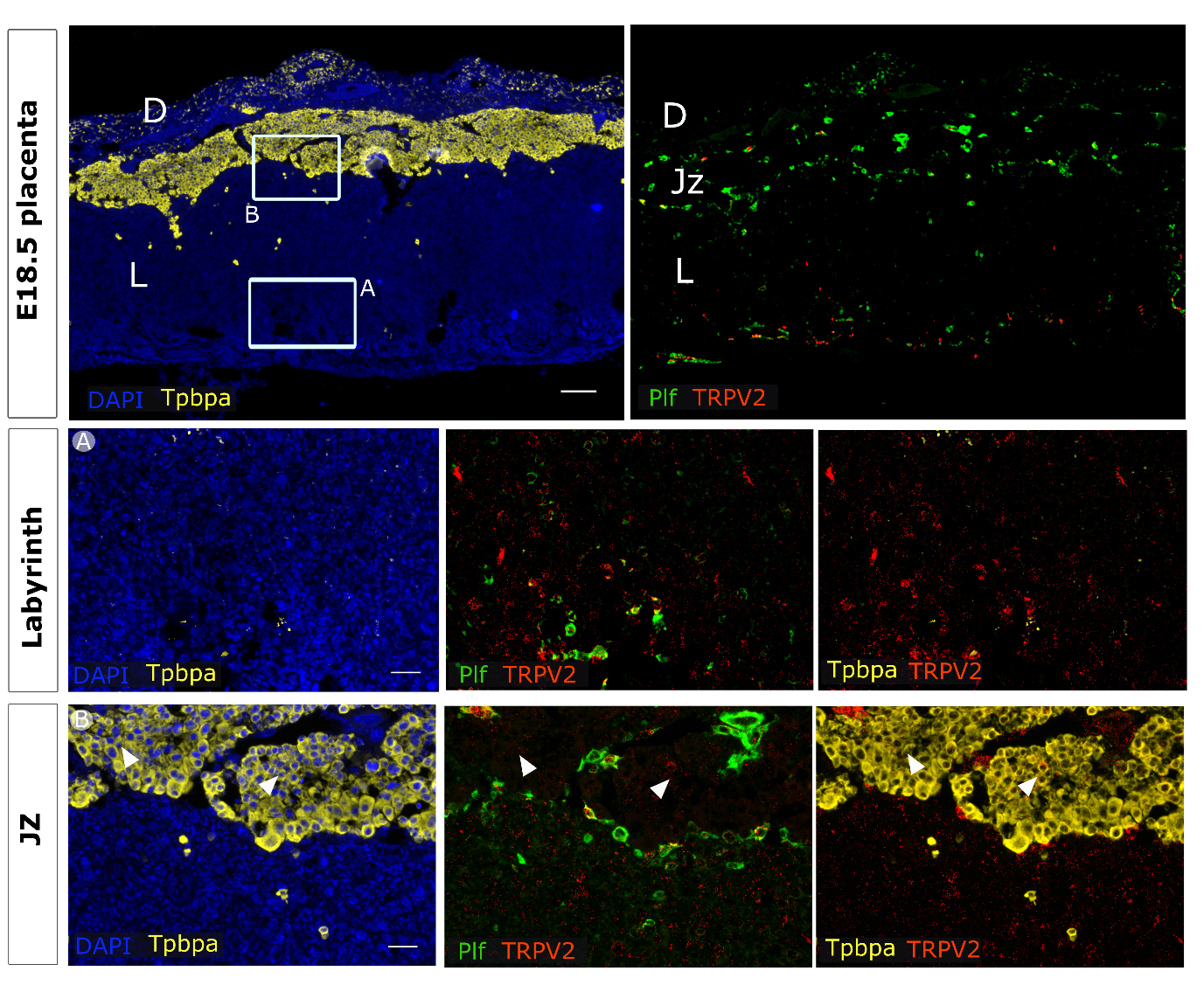
**

**Supplementary Fig. 3 *Trpv2* expression in term placentas**

mRNA *in situ* hybridisation of *Trpv2*, *Tpbpa* as a marker for the junctional zone, *Plf* as a marker for TGCs. An overview of the E18.5 placenta is shown (scale bare = 200 µm). Magnifications of inserts A (Jz) and B (Labyrinth) are presented below (Scale bar = 50 µm). Dapi was used for nuclear staining. Arrowheads point to subtle expression of *Trpv2* in some *Tpbpa*^+^ spongiotrophoblast cells. In A, a *Tpbpa^-^/Plf*^+^ giant cells of the junctional zone is circled. L = Labyrinth, Jz = Junctional zone, D = Decidua, Tpbpa = Trophoblast-specific protein alpha, Plf = proliferin.

**
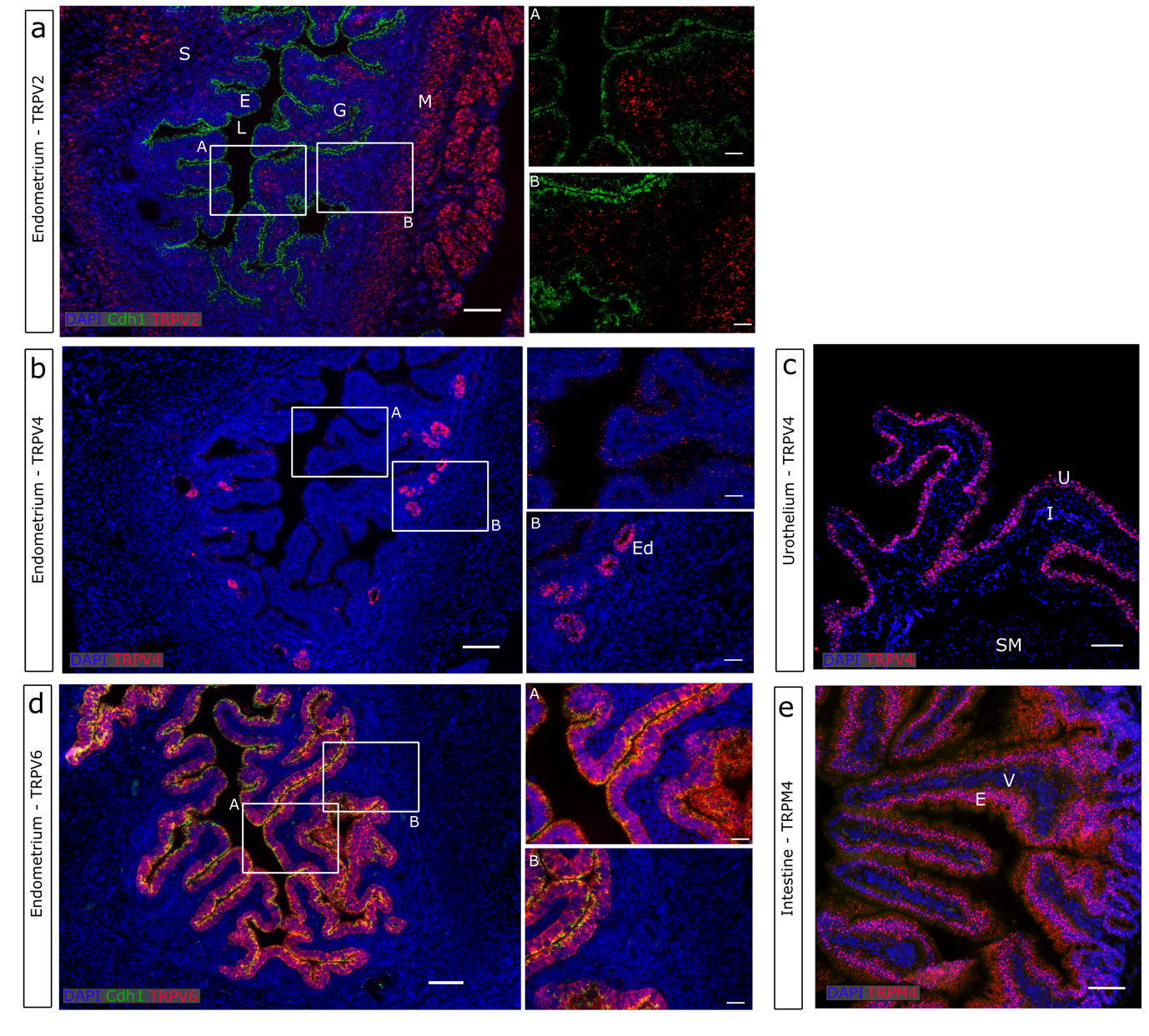
**

**Supplementary Fig. 4 positive control staining**

(a) In line with literature, *Trpv2* and *Cdh1* are mutually exclusive suggesting *Trpv2* is mainly expressed in the stroma. (b) *Trpv4* expression is observed in the epithelium as well, but is more strongly expressed in cells that are likely *Cdh1^-^* vascular endothelial cells. (c) *Trpv4* expression in bladder urothelium. (d) *Trpv6* and *Cdh1* are co-expressed and exclusive to the epithelium. (e) *Trpm4* in epithelial cells of the intestines. DAPI was used as nuclear staining. Scale bar = 200 µm for overview and 25 µm for magnification. Insert A = uterine lumen, Insert B = stroma. Cdh1 = E-cadherin, S = stroma, E = epithelium, L = uterine lumen, G = endometrial gland, M = myometrium, Ed = endothelial cell, U = multi-layered urothelium, I = sub-urothelial interstitial cells, SM = smooth muscle, V = villi


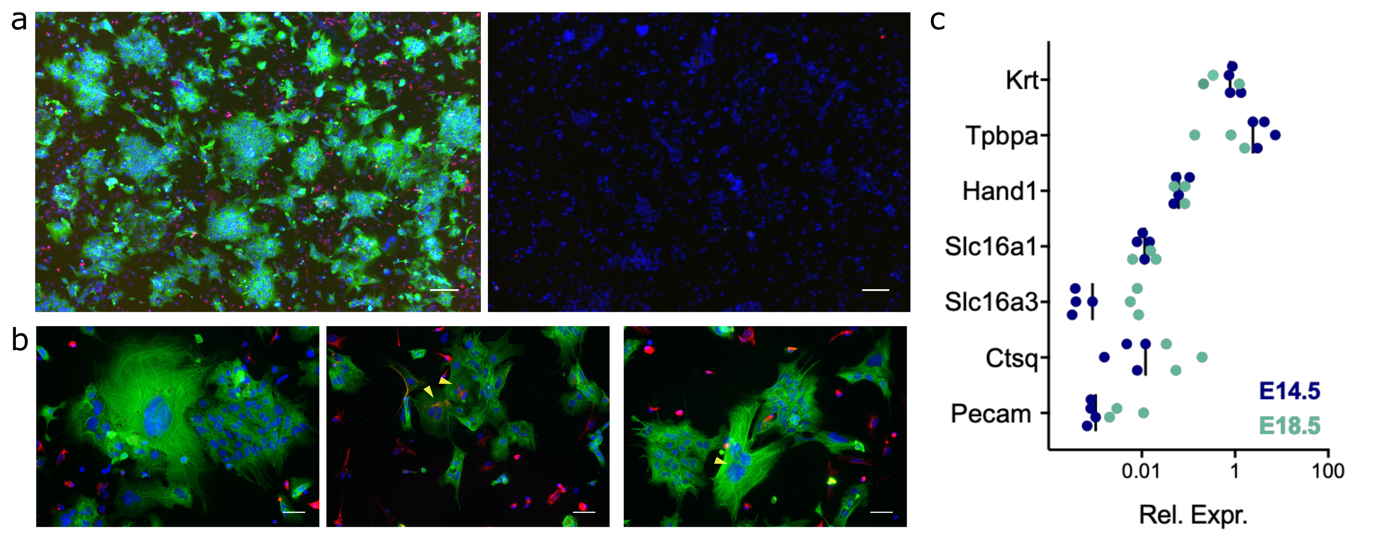


**Supplementary Fig. 5 validation primary trophoblast cells**

(a) Left panel: Immunocytochemistry of primary mouse trophoblasts at E14.5 of gestation of Cytokeratin (green), marker of all trophoblast cells, and Vimentin (red), marker of mesenchymal cells. Right panel: negative control by omitting the primary antibodies. Scale. Bar = 200 µm. (b) images of specific trophoblast morphology visualized with Cytokeratin/vimentin double staining. Left: giant cell, middle and right: multinucleated cells (arrow head). Scale bar = 50 µm. (c) Relative expression of trophoblast markers in primary cell cultures at E14.5 (n=3) and E18.5 (n=3), relative to the geometric mean of housekeeping genes *Gapdh* and *Actb*.

**
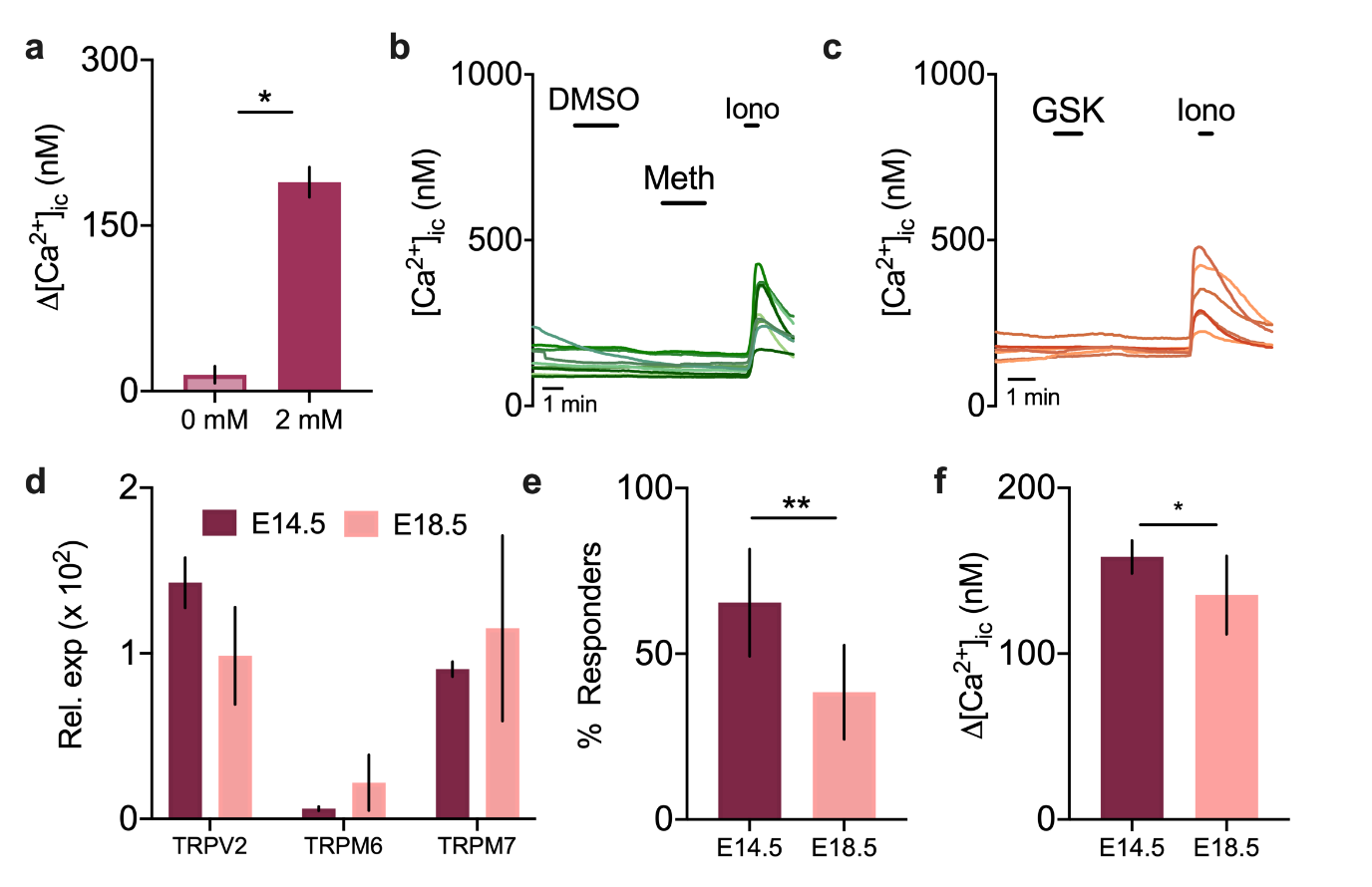
**

**Supplementary Fig. 6 Additional evidence for TRP channel expression**

(a) Calcium amplitude during application of THC while calcium was omitted from the extracellular solution, and during application of THC in extracellular solution with 2 mM calcium. (b) Example traces of intracellular calcium during application of vehicles (0.1% DMSO for ionomycin and GSK016790A, 0.05% Methanol for THC) ; n = 447 cells from 3 experiments. (c) Example traces of intracellular calcium during application of 20 nM GSK016790A ; n= 484 from 3 experiments. (d) Relative expression, assessed with qRT-PCR, of *Trpv2, Trpm6 And Trpm7* in primary cultures isolated at E14.5 (n=4) and E18.5 (n=3), relative to geometric mean of housekeeping genes *Actb* and *Gapdh*. (e) Percentage of responders to THC in primary trophoblast cultures of different gestational days. (f) Intracellular calcium amplitude of cells responding to THC in primary cultures of different gestational days. Statistical differences were assessed with Non-parametric Mann-Whitney test. *: p<0.05, **: p<0.01, n= at least 6 experiments from minimum 3 independent cultures.

**
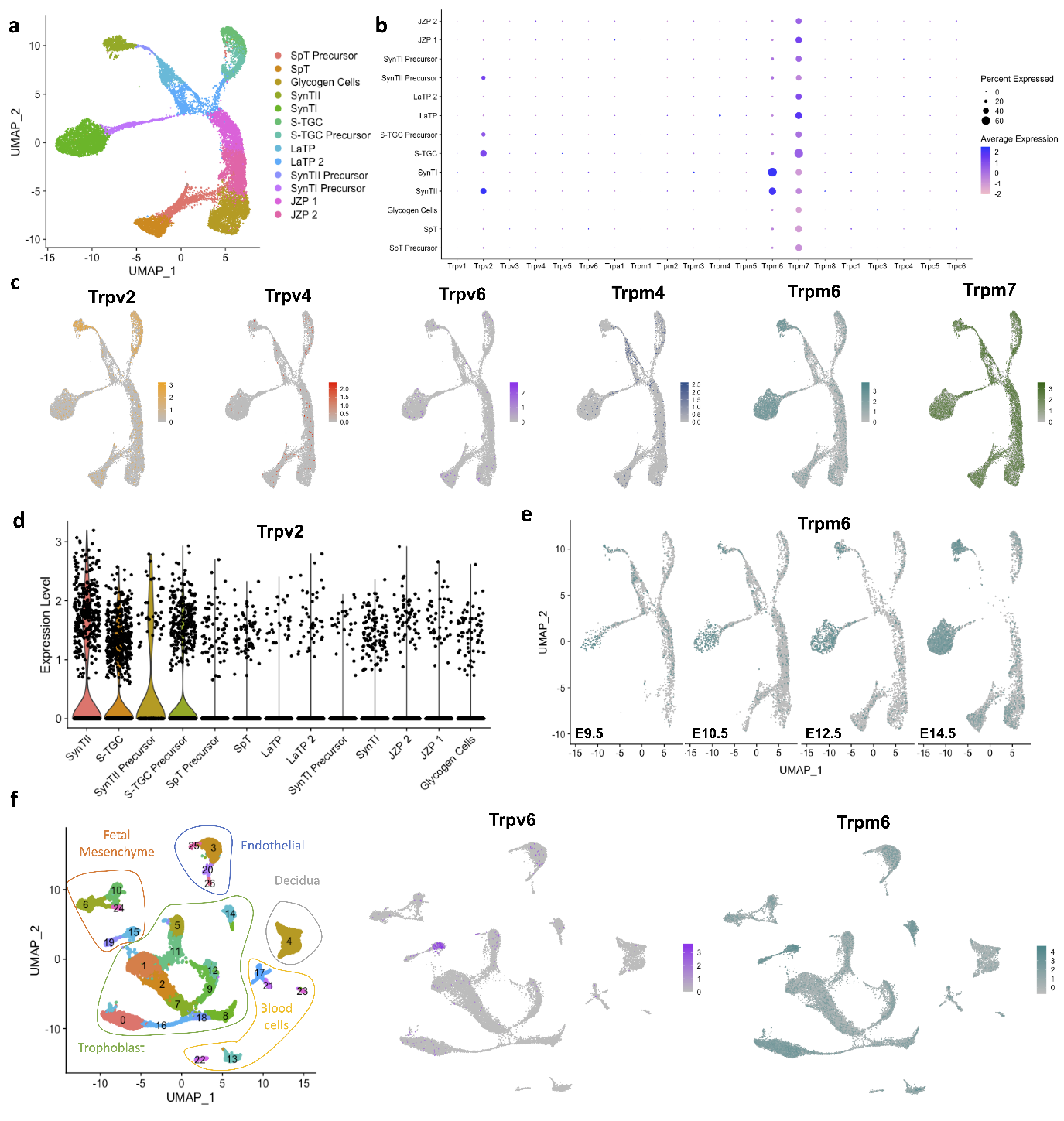
**

**Supplementary Fig. 7 Validation of results**

(a) Uniform manifold approximation and projection (UMAP), allowing for an understanding of the relationship among populations, as visualized by proximity, clustered and plotted according to transcriptome similarity of all trophoblasts. Each dot represents one nucleus colored according to assignment by clustering analysis (b) Dot plot showing average expression and percent of nuclei expressing TRP channels. (c) Expression of identified placental TRP channels in several trophoblast populations projected in UMAP space. (d) Expression of *Trpv2* as violin plots, with nuclei split by cluster identity. (e) Expression of *Trpm6* in UMAP projection split by gestational age. (f) Visualization of all nuclei isolated from the placenta, plotted in two dimensions by transcriptome similarity using UMAP). Each dot represents one nucleus colored according to assignment by clustering analysis. Expression of *Trpv6* and *Trpm6* in all placental cells in UMAP space.
